# Movement can mediate temporal mismatches between resource availability and biological events in host–pathogen interactions

**DOI:** 10.1002/ece3.7478

**Published:** 2021-03-29

**Authors:** Tobias Kürschner, Cédric Scherer, Viktoriia Radchuk, Niels Blaum, Stephanie Kramer‐Schadt

**Affiliations:** ^1^ Department of Ecological Dynamics Leibniz Institute for Zoo and Wildlife Research Berlin Germany; ^2^ Plant Ecology and Nature Conservation University of Potsdam Potsdam Germany; ^3^ Department of Ecology Technische Universität Berlin Berlin Germany

**Keywords:** classical swine fever, dynamic landscapes, global change, host–pathogen dynamics, individual‐based model, movement ecology

## Abstract

Global change is shifting the timing of biological events, leading to temporal mismatches between biological events and resource availability. These temporal mismatches can threaten species’ populations. Importantly, temporal mismatches not only exert strong pressures on the population dynamics of the focal species, but can also lead to substantial changes in pairwise species interactions such as host–pathogen systems. We adapted an established individual‐based model of host–pathogen dynamics. The model describes a viral agent in a social host, while accounting for the host's explicit movement decisions. We aimed to investigate how temporal mismatches between seasonal resource availability and host life‐history events affect host–pathogen coexistence, that is, disease persistence. Seasonal resource fluctuations only increased coexistence probability when in synchrony with the hosts’ biological events. However, a temporal mismatch reduced host–pathogen coexistence, but only marginally. In tandem with an increasing temporal mismatch, our model showed a shift in the spatial distribution of infected hosts. It shifted from an even distribution under synchronous conditions toward the formation of disease hotspots, when host life history and resource availability mismatched completely. The spatial restriction of infected hosts to small hotspots in the landscape initially suggested a lower coexistence probability due to the critical loss of susceptible host individuals within those hotspots. However, the surrounding landscape facilitated demographic rescue through habitat‐dependent movement. Our work demonstrates that the negative effects of temporal mismatches between host resource availability and host life history on host–pathogen coexistence can be reduced through the formation of temporary disease hotspots and host movement decisions, with implications for disease management under disturbances and global change.

## Graphical abstract

Global change is shifting the timing of biological events away from their optima, resulting in temporal mismatches between these biological events and resource availability. We used a spatially explicit individual‐based host–pathogen model to investigate how temporal mismatches of seasonal resource availability and host life‐history events affect host–pathogen coexistence. Our Work demonstrated that host movement decisions and the formations of diseases hotspots can reduce the negative effects of temporal mismatches on host–pathogen coexistence.

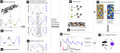

## 1 INTRODUCTION

Environmental fluctuations over time, like diurnal differences in temperature, seasonal changes of climate, or land‐cover modifications due to agricultural practices, can affect species communities in many ways. Many species have adapted to these conditions, so that their biological events match the environmental fluctuations. For example, the onset of mating or breeding (Conaway, 1971), the timing of migration (La Sorte et al., 2015; Mayor et al., 2017), or the timing of prey occurrence (Christian et al., 2007; Sigler et al., 2009) is fundamentally linked to regularly occurring seasonal fluctuations in resource availability such as food or shelter. In many cases, such biological events of species match the regularly occurring changes in the environment, like the onset of spring, because they are triggered by a reliable environmental cue, for example, day length. Mismatches occur when the cue used no longer predicts the timing of the biological event. This mismatch leads to a steady temporal shift of the optimal environmental conditions away from the biological event and can exert strong pressures on population dynamics (Altizer et al., 2006). In marine ecology, mismatches have been found to affect stock recruitment (e.g., for Antarctic krill, Groeneveld et al., 2015). In terrestrial systems, mismatches were demonstrated to increase fitness costs as a result of hatching times (Thomas et al., 2001) and laying dates (Winkler et al., 2002), lagging behind the peak of food availability in seasonally breeding birds (Durant et al., 2007; Schweiger et al., 2008, 2012).

These mismatches affect not only individual species’ performance but also pairwise or multispecies interactions such as the coexistence of a predator and its prey, or a host and its pathogen (Hossack et al., 2013; Kharouba et al., 2018; Mayor et al., 2017; Tonkin et al., 2017). With many species being unable to adapt quickly enough—if at all— to a shift of environmental conditions (Bellard et al., 2012; Radchuk et al., 2019; Visser, 2008), it becomes increasingly important to understand the long‐term community consequences for interacting species under global change.

Within‐year seasonality is one of the strongest and most‐studied forms of periodically occurring environmental fluctuation affecting communities. Seasonality can be defined as an annually reoccurring change of one or more abiotic variables, such as temperature or precipitation (Kharin et al., 2013). These naturally occurring fluctuations are characterized by a positive autocorrelation, meaning that the closer measurements are in time, the more similar will they be on average compared to temporally distant measurements (Dornelas et al., 2013; Koenig, 1999; Legendre, 1993). Temporal within‐year seasonality is similar in its effects to spatial heterogeneity within landscapes as it creates temporary niches of varying levels of resource availability (Tonkin et al., 2017; Williams et al., 2017). While both temporal and spatial fluctuations can have stronger or weaker effects by themselves, they generally work in concert (Durant, 1998), leading to spatiotemporal autocorrelation in resources availability within years.

This spatiotemporal autocorrelation in the environment leads resource levels to vary across the year, and may increase population density when resource availability is highly coincident with a biological event, for example, the timing of birth peaks (Altizer et al., 2006; van Moorter et al., 2013). In this case, environmental and biological events are synchronized. Drivers like global change (e.g., climate or land‐use change) can increase the mismatch between resource availability and timing of the biological event (Durant et al., 2007). A subsequent decline in population size could lead to a decreased coexistence of directly affected and any dependent species. In contrast, such an asynchronous temporal resource availability, if occurring on landscapes with heterogeneous resource availability, could offset the negative effect of the temporal mismatch on coexistence by creating local patches with suitable conditions. This could further lead to a metacommunity‐like structure with increased metacommunity persistence (Duncan et al., 2013).

We here use host–pathogen interactions as a model system to explore the consequences of temporal mismatch on disease dynamics under global change. Global change increasingly affects the phenology of resources, with ensuing consequences for the host's life history and its large‐scale movements and effects of pathogens on host survival and reproduction on the other hand (Semenza & Menne, 2009; Semenza & Suk, 2018).

Climactic fluctuations have triggered outbreaks and facilitated range shifts in pathogens such as West Nile virus, Zika virus, Borrelia bacteria, or other tick‐borne pathogens (Marcantonio et al., 2015; Ostfeld & Brunner, 2015; Semenza & Suk, 2018, see also review in Altizeret al., 2013).

This highlights the importance of environmental conditions in understanding disease dynamics. In this context, resource variation is an essential driver of the distribution of individuals within a host population over space and time. The transmission of many infectious agents depends on direct contact between infected and susceptible hosts, mediated by their movement decisions (Tracey et al., 2014). Hence, understanding how host–pathogen interactions are affected by mismatches on a local spatial and temporal scale is important to implement preventive strategies and develop predictive models.

While there have been studies tackling the effect of landscape heterogeneity on pathogen transmission where limited high‐resource areas can lead to transmission hotspots (Benavides et al., 2012; Nunn et al., 2014) as well as studies considering individual movement (Lane‐deGraaf et al., 2013; Scherer et al., 2020; Tracey et al., 2014), very few studies take asynchronous effects between resource levels and host life‐history events into consideration. Additionally, these few exceptions mainly focus on the effects on vector lifecycles, for example, for ticks and mosquitoes (Estrada‐Peña et al., 2014; Wang et al., 2016). Hence, there is a lack of theoretical studies linking the direct and indirect effects of global change‐induced temporal mismatches on host–pathogen coexistence and dynamics through multiple scales, for example, spatial and temporal heterogeneity in resource availability and individual host movement (Meentemeyer et al., 2012; Rees et al., 2013; White et al., 2018a).

Mechanisms underlying such a mismatch could lead to an increase, but also to a decrease in host–pathogen coexistence: On the one hand, when applying autocorrelated temporal resource dynamics to a spatially heterogeneous landscape, transmission hotspots could form in areas that have higher resource availability than the surrounding landscape. This would facilitate pathogen persistence in those hotspots and subsequently enable the pathogen to be transferred back to other host subpopulations after they have recuperated from low resource conditions (Duncan et al., 2013). On the other hand, if the resource availability changes randomly, meaning there is neither spatial nor temporal correlation in resource availability, the reduction of resources can lead to an immediate and severe drop in host density. Subsequently, such a drop in host density could lead to pathogen extinction. (Altizer et al., 2006; Tonkin et al., 2017).

**FIGURE 1:**
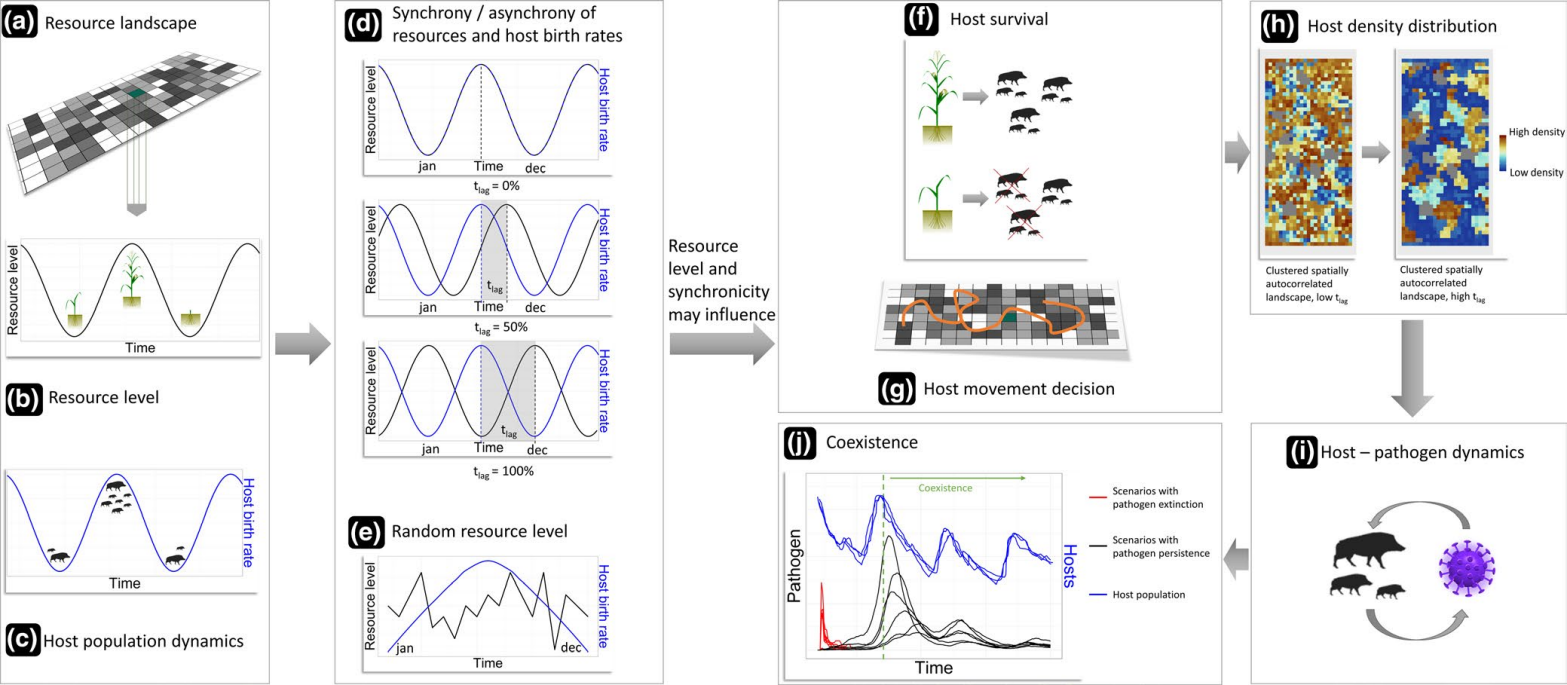
Cascading effect from the resource landscape (a) through the dynamic resource level of each single habitat cell (b) and the host population dynamics (c) that can be synchronous, asynchronous (shifted by t_lag_) (d), or random (e) in time, respectively, to each other. The resource level at specific points in time may influence host survival (f) and movement decisions (g) that may alter host population density distribution (h) and subsequently host–pathogen interactions through contact rates and transmission (i) before ultimately accentuating scenarios that allow for coexistence (j)

We investigated the effect of a temporal lag in resource availability leading to a temporal mismatch between resource availability and the host reproduction probability on pathogen persistence. Asynchrony between the resource availability and the host reproduction probability effectively creates a cascading effect from the resource landscape through host survival, host movement decisions, and resulting host density to pathogen transmission and survival (Figure 1). We study this propagating effect of a temporal mismatch in a bottom‐up driven, interdependent system where the pathogen is dependent on the host, which in turn is dependent on the resource. In detail, we investigate how a constantly shifting temporal lag in peak resource availability away from the timing of host birth events affects host–pathogen coexistence. To this end, we used a modified version of an existing spatially explicit individual‐based host–pathogen model of a group‐living social herbivore, that is, classical swine fever (CSF) virus in wild boar (*Sus scrofa*) (Kramer‐Schadt et al., 2009; Scherer et al., 2020).

We hypothesized that a temporal mismatch alters disease dynamics depending on the intensity of the mismatch between environmental resource availability and host life‐history events and that movement can mediate or reverse the effects of asynchrony. In accordance with theory, we expect that unpredictable random changes in host resource availability over time, for example, induced by agricultural land‐use practices like harvesting (Ullmann et al., 2018, 2020) or hunting, result in low coexistence probability due to increasing chance events, leading to higher disease extinction (Melbourne & Hastings, 2008). In contrast, seasonality increases coexistence probability (Altizer et al., 2006). However, increasing asynchrony between seasonal resource availability and host life‐history events will lead to a decrease in host–pathogen coexistence. Movement can reverse these processes by bridging spatiotemporal troughs in local host density, thereby increasing disease persistence. We discuss our results in terms of consequences for disease persistence under climate and land‐use change conditions that may be provoked by increasing asynchrony of relevant time scales.

## 2 METHODS

### 2.1 Model overview

We used a spatially explicit individual‐based eco‐epidemiological model developed by Scherer et al., (2020). It is based on earlier models only considering neighborhood infections developed by Kramer‐Schadt et al., (2009) and Lange et al., (2012, 2012). The model by Scherer et al., (2020) relies on individual movement decisions of host individuals, that is, long‐distance roaming movement of males (hereafter termed “movement”), a process important for disease transmission. We further modified the model by adding spatiotemporal landscape dynamics representing changing resource availability, a response of movement decisions to that landscape and a resource‐based mortality. A complete and detailed model description following the modified ODD (Overview, Design concepts, Detail) protocol (Grimm et al., 2006, 2020) is provided in the supplementary material, and the model implementation is available in the Zenodo Database (Kürschner et al., 2021).

Overall, the model comprises two main components, a host life‐history model and an epidemiological pathogen model. Host individuals are characterized by sex, age, location, demographic status (residential, group split of subadults and resource‐based displacement to the neighboring cells, and male long‐distance roaming movement), and an epidemiological status. The latter is defined by an SIR epidemiological classification (susceptible, infected, and recovered; Kermack & McKendrick, 1927). Recovered individuals gain lifelong immunity and can pass on temporary immunity via maternal antibodies to their offspring. The pathogen model alters host survival rates, reproductive success, and infection length given its virulence.

### 2.2 Landscape structure

The landscape structure is comprised of a spatial grid of 1,250 2 km × 2 km cells each representing the average home range of a wild boar group (Kramer‐Schadt et al., 2009), totaling a 100 km × 50 km landscape. The landscape is a self‐contained system without any outside interaction or movement beyond the landscape border. Each cell is characterized by a variable resource availability (habitat quality) that is expressed as female host breeding capacity and that translates directly into possible group size, with the minimum being one breeding female per group to a maximum of nine. The initial resource availability was calibrated to achieve the reported average wild boar density of five breeding females per km^2^ (Howells & Edwards‐Jones, 1997; Melis et al., 2006; Sodeikat & Pohlmeyer, 2003). We investigated several landscape scenarios of varying spatial complexity, ranging from a fully random landscape structure to different degrees of random landscape clusters generated in R (R Core team, 2019) using the NLMR package (Sciaini et al., 2018) while keeping the mean female breeding capacity constant at 4.5 females across the different landscapes, where all landscape cells, including the ones that are not suitable as habitat, are considered (Supplementary material Appendix Figure S4).

The spatiotemporal landscape dynamics are superimposed on the different types of landscapes, and the dynamics are designed to mimic seasonal changes by gradually increasing and decreasing resource availability. Resource availability in each cell increases in 5‐week intervals for approximately 25 weeks from the beginning of the year and then declines in 5‐week intervals for the following 25 weeks. Resource availability translates directly into the breeding capacity for each cell and cannot, during the increase, exceed the maximum breeding capacity of nine females and cannot decrease below one female during the decrease period. A breeding capacity < 1 could lead to inflated extinction scenarios, depending on the clustering of the landscape, through the creation of artificial barriers that would isolate host groups and prevent the pathogen from being spread. In case of wild boar, the increase or decrease of resource availability that occurs periodically throughout the year results in a variation of breeding females with an average over time being 4.5 females supported by one cell. Throughout each simulated year, resource availability changes in parallel to the host reproduction probability (Figure 1d). The resource availability is then temporally shifted (t_lag_) away from the host reproduction probability by 25% increments up to a full mismatch at 100%. A higher level of mismatch reflects an increase of severity in global change. Additionally, we implemented a nonseasonal, unpredictable landscape dynamic, where the resource availability changes randomly (a random integer between one and nine) every five weeks while maintaining a mean of 4.5 throughout the landscape (so‐called “white noise,” Figure 1e).

### 2.3 Process overview and scheduling

The temporal resolution equals the approximate CSF incubation time of one week (Artois et al., 2002). The following procedures were scheduled each step in the following order: pathogen transmission, male host roaming movement, natal host group split of subadult males and females and subsequent resource‐based displacement to the neighborhood, host reproduction, host mortality (disease‐based and resource‐based), host aging, and landscape dynamics. Group split of subadult males and females under no mismatch conditions was limited to week 17 and week 29 of the year, respectively, representing the typical dispersal time for each sex. The order of these procedures was established in previous versions of the models, and changes of the order were not shown to have significant implications to the model outcome.

### 2.4 Main processes

Pathogen transmission—All transmission processes remain unchanged from the model implementation by Scherer et al., (2020). The course of the disease is determined by an age‐specific case fatality rate and an exponentially distributed infectious period for lethally infected individuals. Transient infected hosts have an infectious period of one week and gain lifelong immunity (Dahle & Liess, 1992). Infection dynamics emerge from multiple processes: within group transmission, movement‐based transmission, and individual age‐dependent courses of infection. Within groups, the density‐dependent infection pressure is determined by transmission chance and the number of infectious group members. For roaming males, under movement‐based scenarios, individual per‐step transmission probability is calculated as the transmission rate divided by the movement distance the individual has travelled to account for the time an individual spends in each cell (Scherer et al., 2020).

Male host roaming movement—Our model uses two of the explicit intercell movement rules for males implemented in Scherer et al., (2020) (habitat‐dependent movement: HDM; correlated random walk: CRW) as well as a setup without explicit movement (neighborhood infection). Individuals performing a CRW display a general tendency to continue in the same direction as the previous movement without taking landscape structure or resource availability into consideration (Codling, Plank & Benhamou, 2008; Kareiva & Shigesada, 1983). The decision process of individuals following the HDM rule is related to the underlying landscape directly by tending to move toward landscape cells with higher resource availability. In general, individuals were moving up to an individual weekly maximum movement distance or supplementary rules led to the decision to stay in the current cell. Furthermore, individuals move from cell to cell without within‐cell movement.

Group split of subadults—We implemented two distinct responses to changing resource availability. First, if the theoretical maximum number of individuals in a group is higher than the number of individuals currently present in the group, then the group does not split. However, if low resource availability reduces the theoretical maximum group size below the number of individuals currently in the group, individuals above the current capacity will try to leave the group and establish in an empty neighboring cell based on resource availability. The selection of individuals to leave the group is dependent on the age of the individual, where young individuals will leave the group first.

Host reproduction—Female hosts reproduce once a year, depending on their age class. The number of breeding females is determined by each habitat cell's resource‐dependent breeding capacity. Individual female hosts are checked for their breeding status on a weekly basis to then reproduce depending on the season with a peak in March and no reproduction in winter from October to December.

Host mortality—Another functional response to resource availability is increasing age‐dependent mortality over time. Groups that exceed the theoretical maximum group size have increased mortality depending on the difference between actual group size (number of individuals) and theoretical maximum group size. Furthermore, the maximum survival time for adults in groups above their respective maximum group size was capped at assumed levels between 5 and 20 weeks (for details, see ODD in the supplementary material).

Landscape dynamics with temporal lag—We modeled several levels of temporal lags (t_lag_; Figure 1d). We gradually increased the level from 0% (no change) to 100% (full mismatch between host population dynamics and resource availability) in 25% increments. Each 25% increment represents 5 weeks in the simulation. Therefore, the peak in resource availability is shifted 5 weeks away from the host species reproductive peak in each consecutive increment up to the maximum of approximately 25 weeks. The 25‐week (or 100%) scenario represents the full mismatch of host population dynamics and resource availability. The scenario with 0% t_lag_ was used as control for temporal shift scenarios.

### 2.5 Model analysis

Each simulation was run for 50 years (2,600 weeks) in total, with the virus being randomly released in the second year (weeks 53–104). The virus was introduced to one out of a set of predefined cells in the center of the upper row with a resource availability above the mean of 4.5 during time of release. We ran 25 repetitions per combination of movement rule (3 levels: CRW, HDM, and no roaming movement as a control scenario for movement rules), landscape scenario (4 levels: small clusters, medium clusters, large clusters, and random as a control scenario for the landscape structure), and degree of mismatch (5 levels: t_lag_ 25%, t_lag_ 50%, t_lag_ 75%, t_lag_ 100%, and t_lag_ 0% as a control for mismatch). We analyzed proportional coexistence probability (P_coex_) estimated over each block of 25 repetitions by counting the times both host and pathogen survived during the simulations. Furthermore, for simulations where coexistence was not achieved, we measured the time to pathogen extinction (t_ext_). Due to the spatial variability of clusters throughout the landscape, the overall densities of hosts and pathogens varied too little across the different landscapes and scenarios to provide more detailed insight, while measuring local per‐cell densities was beyond the scope of the study. Therefore, we also analyzed the spatiotemporal distribution of infected hosts in the landscape by recording the number of timesteps an infected host was present in each landscape cell. Next, we applied the autocorrelation function (acf) at lag 2 to the frequency distribution of the cumulative time the pathogen was present in each landscape cell, so as to characterize the amount of time and proportion of the landscape with pathogen presence in the different scenarios. The higher the value of acf, the more similar is the cumulative time with pathogen presence across all the cells in the landscape. On the contrary, a low acf at the following timestep (lag 2) indicates that the cumulative time of pathogen presence differs among the cells across the landscape, more precisely, that very few cells have the pathogen present, whereas the majority of cells never has an infected host present.

## 3 RESULTS

### 3.1 Host–pathogen coexistence and disease persistence

**FIGURE 2:**
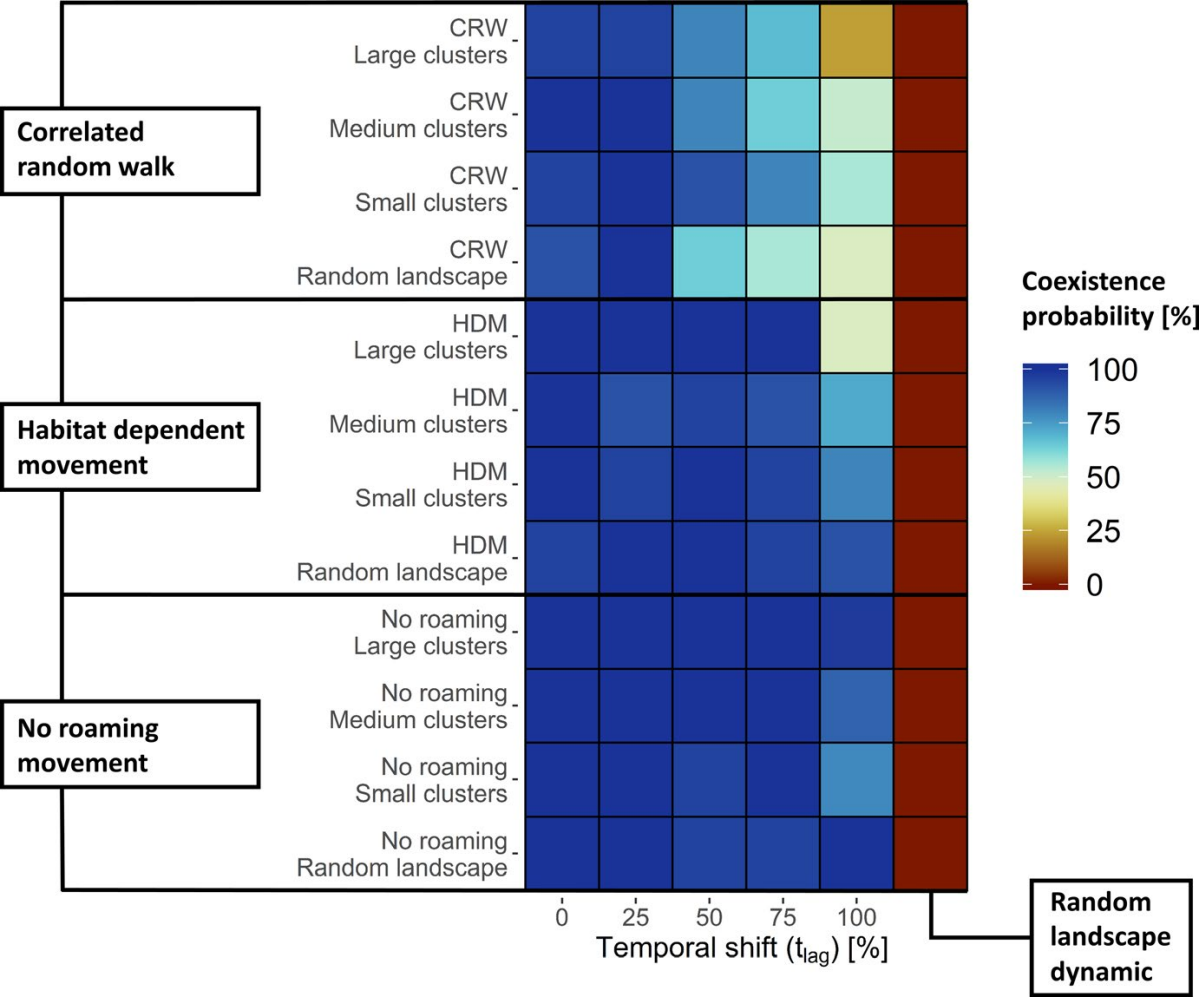
Coexistence probability (P_coex_) estimated as the proportion of simulation runs in which both host and pathogen survived (color gradient). P_coex_ is grouped by three types of applied movement rules (correlated random walk—CRW, habitat‐dependent movement—HDM, and no roaming movement (control)) and four different landscape configurations (large, medium, and small clusters and random configuration) along an increasing temporal mismatch (0%–100%) including a random dynamic (control)

The coexistence probability (P_coex_) was lower in the two scenarios with roaming movement compared to the movement control (scenarios without roaming movement, Figure 2). Importantly, in both scenarios with movement the coexistence also decreased with increasing temporal mismatch (t_lag_) and increasing landscape homogeneity (large clusters). That means, in contrast to our predictions, movement decreased coexistence or pathogen persistence. The decrease in P_coex_ was, however, more severe in scenarios with random movement (CRW) where P_coex_ at t_lag_ 100% decreased to 24% from 96% in the control (t_lag_ 0%; i.e., no mismatch) in large‐cluster landscapes compared to a P_coex_ decrease to 48% from 92% in the control in similar scenarios with habitat‐dependent movement (HDM). On average, over all landscape configurations, P_coex_ at t_lag_ 100% decreased to 45% with CRW, 73% with HDM and 91% in the movement control. In general, the control for temporal mismatch showed a P_coex_ between 100% and 92% for CRW, between 100% and 96% for HDM, and 100% in movement control scenarios (no roaming movement). Random landscape dynamics yielded a P_coex_ of 0% in all landscape and movement scenarios.

### 3.2 Pathogen extinction time

**FIGURE 3:**
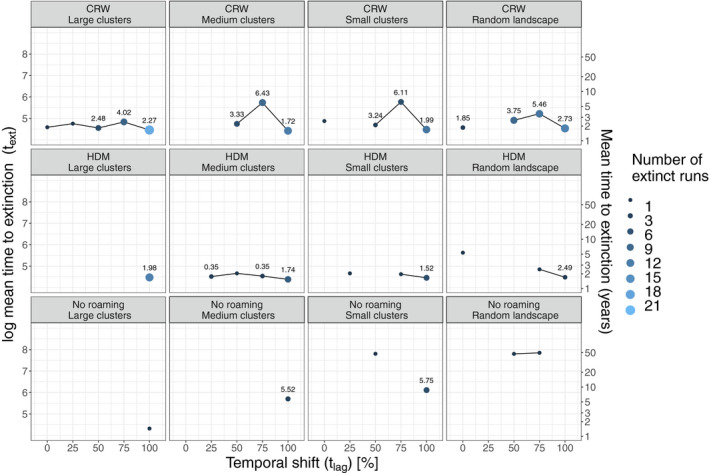
Mean log pathogen extinction times for all simulation scenarios where the pathogen went extinct separated for movement scenarios (CRW—correlated random walk, HDM—habitat‐dependent movement, and no roaming movement (control)), landscape configuration, and temporal shift (t_lag_). The number of extinct runs (gradient and size) is relative to the 25 total runs that were conducted per combination of movement, landscape, and t_lag_. The number above the points is the standard deviation of the log mean pathogen extinction time, where applicable

We assessed the mean pathogen extinction time for simulations with P_coex_ below 100% (Figure 3). For most scenarios, the pathogen went extinct around the time of pathogen release. As an exception to that, the t_lag_ 75% scenarios with CRW movement had a higher t_ext_ than the other scenarios. Notably, for both types of movement (CRW and HDM) t_ext_ was shorter at t_lag_ 100% when compared with the other t_lag_ scenarios. That means, very restricted or directed movement as in the HDM does considerably delay pathogen extinction to high t_lag_ scenarios. Early onset of many disease clusters, as with CRW, again synchronized the outbreak temporally across the landscape, leaving no high‐density host cluster behind for bridging infections especially when the host peak density is completely mismatching peaks in resource availability (i.e., t_lag_ = 100%).

### 3.3 Spatial patterns in coexistence

**FIGURE 4:**
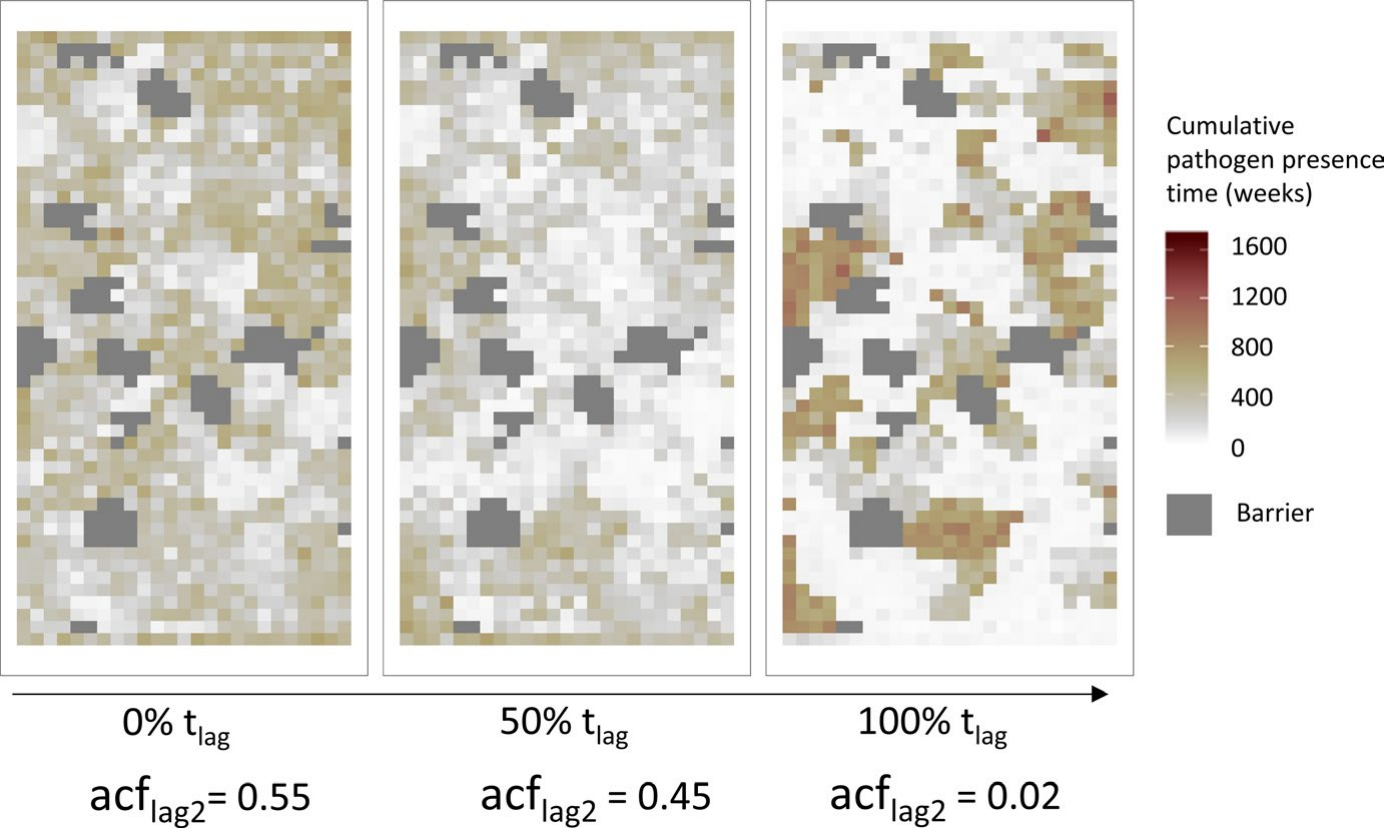
Spatial distribution of infected hosts in the correlated random walk movement scenario (CRW) applied to a landscape with medium clusters. The color gradient shows the cumulative pathogen presence time in weeks, that is, how long the pathogen was present in a landscape cell including the acf (autocorrelation function) values at lag 2 for those scenarios. Each frame represents a single representative example run with increasing temporal shift (t_lag_) from left to right

**FIGURE 5:**
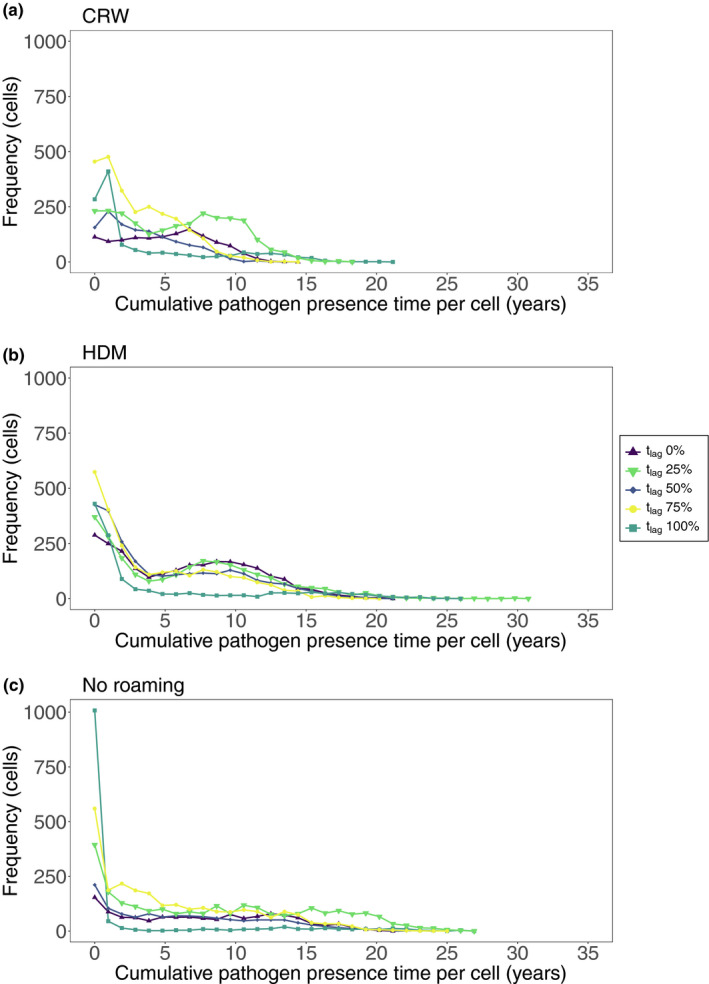
Frequency of cells with infected hosts throughout simulation runs with CRW movement (a), HDM movement (b), and no roaming movement (c) for all five t_lag_ scenarios. The cumulative pathogen presence time per landscape cell represents the total amount of time that a landscape cell had infected hosts occupying it, while frequency is the overall number of cells with the same pathogen presence time values at the end of the simulation

To assess the effects of resource availability on host survival, host movement, and pathogen survival, we explored the spatial distribution of infected hosts over the course of the simulations where coexistence was achieved. We found highly similar spatial patterns across all landscape scenarios, and thus, we here use the medium‐cluster landscape as an example case in the following. For scenarios using the CRW movement rule, we saw a decrease in acf (autocorrelation function) at lag 2 with increasing t_lag_ from 0.55 at t_lag_ 0% to 0.45 at t_lag_ 50% (Table 1). This decrease in acf means that with an increasing t_lag,_ there were fewer cells with similar cumulative time of pathogen presence, that is, the cumulative time with pathogen presence differed increasingly among the cells. Such a difference among the cells in the cumulative time with pathogen presence increased further for the scenarios with t_lag_ 100% as indicated by acf 0.02, meaning that only a small fraction of the landscape carried the infected hosts, but a large number of the grid cells were either never infected or only for short periods of time. Figure 4 shows an example for the spatial clustering for the CRW movement scenario; more detailed figures for all movement and landscape scenarios can be found in supplementary material Appendix Figures S5–S7. The frequency distribution of the cumulative time the pathogen was present in each landscape cell (Figure 5a) indicates that, in scenarios with t_lag_ 75 and 100% compared to the scenarios with low t_lag_, the infected hosts were present in only a small fraction of the landscape for an extended period. In most of the other parts of the landscape, infected hosts were only present for a short duration or were not present at all.

**TABLE 1:** Acf (autocorrelation function) values at lag 2 for all movement scenarios (CRW—correlated random walk, HDM—habitat‐dependent movement, and no roaming movement (control)), and temporal shift (t_lag_) combinations including the 95% confidence interval (ci)

Movement	t_lag_	ci±	acf lag 2
CRW	0	0.51	0.55
	25	0.46	0.49
	50	0.54	0.45
	75	0.51	0.32
	100	0.60	0.02
HDM	0	0.60	0.19
	25	0.49	0.25
	50	0.60	0.09
	75	0.60	0.13
	100	0.52	0.01
No roaming	0	0.60	0.12
	25	0.50	0.24
	50	0.54	0.34
	75	0.54	0.24
	100	0.52	−0.02

Within HDM scenarios (Table 1), while there was a lower acf at t_lag_ 100% (0.01) compared to t_lag_ 0% (0.19), there was no steady decrease in acf with increasing t_lag_ as found for CRW scenarios. Interestingly, however, the acf for HDM scenarios was on average lower than for CRW scenarios, indicating that under the HDM scenario, the number of cells with a similar cumulative time of pathogen presence was lower than under the CRW. The frequency distributions of the cumulative time the pathogen was present in each landscape cell for HDM scenarios (Figure 5b) showed similar patterns to those found for CRW: In scenarios with high t_lag,_ the pathogen was either absent from the majority of the cells or present in a small fraction of the landscape.

Scenarios without roaming movement (control, Figure 5c) also showed the lowest acf at t_lag_ 100% with −0.02 when compared to all other t_lag_ scenarios (Table 1). The acf at t_lag_ 50% (0.34) was, however, higher than the acf at t_lag_ 75% (0.24), indicating that at this intermediate time lag, we find the highest number of cells (i.e., larger proportion of the landscape) with similar cumulative time of pathogen presence. The t_lag_ 100% scenario showed 940 out of 1,250 cells without infected hosts throughout the entire simulation and thus had no infected hosts in a large portion of the landscape while still having an 88% P_coex_.

## 4 DISCUSSION

While previous modeling approaches have theoretically demonstrated the importance of interactions between landscape structure, individual movement behavior, and pathogen transmission for predicting and understanding disease dynamics (Scherer et al., 2020; White et al., 2018b), few studies have addressed how an increasing asynchrony between resource availability and dependent host biological processes influences host–pathogen coexistence. Given the current climate warming crisis with increased mismatch between resource availability and host phenological events (Plard et al., 2014; Post & Forchhammer, 2008; Visser & Gienapp, 2019), the knowledge on how disease dynamics might toss and turn in the future is of utmost importance for managing emerging and zoonotic diseases (White et al., 2018b). Also, tree mast years (Doublet et al., 2019) or land‐use practices like harvesting, crop rotation, or asynchronous anthropogenic pressures such as hunting might lead to mismatches. While climate warming might induce mismatch effects negatively affecting host life history, climate warming might have direct effects on the pathogen, too, amplifying either positive or negative consequences on the host. Indeed, it is reasonable to expect that some diseases will adapt to changing environmental conditions and potentially increase in prevalence (Rohr & Cohen, 2020; Thomas, 2020). However, here we focus on mismatch effects on host life history.

In accordance with theory, we found that random fluctuations of resources decreased coexistence, whereas occurrence of biological events in synchrony with resource seasonality increased coexistence (Altizer et al., 2006; Heino et al., 2000; Roy et al., 2005; Wichmann et al., 2003). This is underpinned by studies demonstrating that seasonality, for example, in transmission rates, can alter the dynamics of host–pathogen interaction and feed back to effects on the hosts density as demonstrated by Bolzoni et al., (2008) for rabies in several species. Other studies found similarly complex dynamics arising by applying seasonal dynamics to the host birth rate as done by Ireland et al., (2007) for rabies in foxes. However, for full mismatch conditions, our model yielded still rather high host–pathogen coexistence probabilities, contrary to our initial expectations.

*Disease hotspots resulting from spatiotemporal asynchrony*—One apparent factor for coexistence despite completely decoupled environmental and biological events, throughout all simulations, was the spatiotemporal clustering of infection hotspots with increasing mismatch, forming disease islands in the landscape. The emergence of disease islands due to asynchronous resource dynamics has also been shown experimentally by Duncan et al., (2013) and theoretically by Becker and Hall (2016). The spatial restrictions to relatively small infection hotspots suggest a lower coexistence probability, with small host populations not being able to sustain a prolonged disease outbreak. Usually, a disease outbreak within an island can lead to critical loss of host individuals up to the point where the population cannot recuperate on its own (Clifford et al., 2006; Walker et al., 2008). However, in our case those hotspots were not constantly isolated from each other or the surrounding landscape. In this case, theory predicts that asynchrony in resource availability throughout the landscape facilitates demographic rescue by movement or migration (Roy et al., 2005). Our model demonstrated this effect that subsidizes pathogen persistence and thus coexistence even when the timing of resource scarcity coincided with the seasonal reproduction peak at a t_lag_ of 100%. Simulations of this “worst‐case scenario” of resource mismatch showed that high‐resource cells within the landscape that can support a higher population density were constantly recolonized if the pathogen depleted the host population in some of those cells during low resource conditions. On the other hand, during “high‐resource periods,” the entire remaining population could spread more evenly throughout the landscape. High‐resource habitat clusters where the pathogen went temporarily extinct and thus harbored largely undisturbed host populations can function as partial refuges for many host individuals. Once the resource availability in the surrounding habitat becomes more favorable, these individuals can spread out and recolonize potentially depleted habitat cells and could subsequently come into contact with infected hosts. Respectively, high‐resource habitat clusters where the pathogen was constantly present function as partial sources for the pathogen allowing it to be reintroduced into now susceptible or naïve subpopulations (Elkin & Possingham, 2008).

While metapopulation dynamics and source–sink dynamics have been well studied and documented (Bansaye & Lambert, 2013; Foppen et al., 2000; Nagatani et al., 2018), including the effect of temporal autocorrelation (Gonzalez & Holt, 2002; Roy et al., 2005), our model showed a metacommunity structure only under temporal mismatch scenarios. This reduces any negative effect a mismatch could have on coexistence, such as pathogen extinction through critically low host density. Only when the mismatch increased above 50%, did the distribution of infected hosts start to aggregate in certain parts of the landscape, forming a metacommunity structure with the pathogen.

Host density and connectivity are the two most important factors that determine contact rates and subsequent disease transmission in directly transmitted diseases (Parratt et al., 2016). The timing of the initial outbreak was variable between a burn‐in phase of one year and the third year. This temporal variability of starting the initial outbreak might have led to the fact that in some simulations, the pathogen could never establish and invade the host population, leading to many extinction times shortly after pathogen introduction. This was especially prominent in mismatch scenarios. However, when the pathogen was able to establish in the host population beyond the initial outbreak and subsequently spread through the landscape, extinction became increasingly unlikely. The increase in early extinctions with increasing mismatch further emphasizes an increasing importance of the timing of biological events. Pathogens are less likely to cause a widespread disease outbreak when being introduced into a susceptible population during a period of unfavorable environmental conditions.

*Movement and decision‐making in animals as key mechanism of coexistence*—Spatially explicit host movement effectively mitigates some of the spatiotemporal restrictions a dynamic resource landscape can put on host and/or pathogen. As Scherer et al., (2020) have shown for static landscapes, explicit host movement can increase coexistence, and this also holds true for many instances within a spatiotemporally dynamic landscape. However, movement is also subject to a larger variability, for example, in distance moved or in timing of movement and can, in fact, become detrimental to coexistence under certain circumstances. There is, for example, the possibility that an infected host individual transports the pathogen to susceptible populations that have not fully recuperated from unfavorable habitat conditions and lack the population density to sustain the pathogen. This is especially prominent for nondirected movement such as correlated random walks, neglecting important species–landscape interactions (White et al., 2018b).

Particularly under random movement, there is a lower chance, compared to directed habitat‐dependent movement, for infected host individuals to transmit the disease to susceptible hosts in high resource, high host density habitat clusters fast enough to allow for pathogen persistence. This is not apparent under habitat‐dependent movement, due to directed movement toward high‐resource areas. In consequence, under the directed habitat‐dependent movement, the chance of coming into contact and infecting other hosts increases, while moving toward or through high‐resource habitats. Hence, movement‐induced species interactions could be a key mechanism promoting disease persistence, which is underpinned by studies on waterfowl (Figuerola & Green, 2000) or white‐fronted geese (Kleijn et al., 2010). Also, White et al., (2018b) have demonstrated theoretically that decision‐making of animals, here the decision to move toward high‐quality habitat, might increase disease persistence.

*The effect of landscape structure on coexistence*—An increasing homogenization of the landscape particularly through anthropogenic land‐use change, for example, deforestation or the increase in agricultural areas (Patz et al., 2004), can, however, offset the pronounced effect of habitat‐dependent movement on coexistence. Individuals within large clusters, comprised of the same level of resources, might not be able to find higher resource areas in time. In addition, a large proportion of the host population is situated in similar habitats and subjected to the same level of resource decrease simultaneously. Consequently, the loss of susceptible hosts due to death or immunization cannot be compensated with the influx of new susceptible hosts, for example, though birth or immigration, to sustain the pathogen (McCallum, 2012), causing it to go extinct in large parts of the landscape. Accordingly, our results demonstrate lower coexistence in landscapes with larger homogeneous habitat clusters.

Interestingly, while increasing spatial homogeneity of the landscape had a negative effect on pathogen persistence, we still found the formation of infection hotspots in scenarios with full temporal mismatch between host reproduction and resource peaks. Infection hotspots were, with varying degree, present across all tested landscape configurations in scenarios with full mismatch and across all tested movement scenarios. While the initial spatial resource structure might not be important when it comes to the emergence of infection hotspots, larger low‐resource areas in more homogeneous landscape configurations can form temporary barriers, similar to seasonal landscape barriers (Mui et al., 2017). These temporary landscape barriers can restrict the pathogen to certain areas in scenarios where host individuals have no explicit long‐distance movement (Supplementary material Appendix Figure S7). While there was no strong effect of these temporary barriers on coexistence, this further highlights the role of explicit long‐distance host movement in terms of disease transmission (see review in Altizer et al., 2011).

*A reality check of our model assumptions*—Our individual‐based model here best described a group‐living social animal conducting long‐range movements acting autonomously, that is, deciding on movement directions, while demographic processes are also resource‐dependent. On the other hand, it also considers viral traits like acute to chronic infections in a directly transmitted disease. Thus, our model is quite complex in terms of realistic processes and hence a template for many disease dynamics under temporal mismatch, induced, for example, by climate and land‐use change. The spillover of Hendra virus from flying foxes to other animals (Martin et al., 2015; Plowright et al., 2015) or the impact of the canine distemper virus on spotted hyenas (Benhaiem et al., 2018) or lions (Craft et al., 2011) could be amplified by climate and land‐use change‐induced temporal mismatches.

Climate change is altering environmental fluctuations that lead to increasing mismatches between resources and biological events (Durant et al., 2007). And, although some animal species may adapt to such temporal shifts in resource availability, they might respond too slowly to be able to persist (Radchuk et al., 2019). Yet, our results demonstrate that we could expect the emergence of disease hotspots under a full temporal mismatch of resource availability and the timing of host birth peaks, counteracting possible adverse effects of reduced host densities. Temporal shifts of the magnitude that were used in this work, that is, large shifts of multiple weeks up to a full mismatch, might not be as important for climate change in the near future, where temporal mismatches are expected to be smaller (Thackeray et al., 2016). However, climate change does not occur separately from other anthropogenically caused threats. Indeed, large‐scale land‐use changes can alter the resource distribution throughout a landscape in more drastic ways resulting in the possibility for stronger mismatches between available resources and host life history (Ullmann et al., ,^2018^, ^2020^). For example, changing natural habitats into agricultural areas could still provide resources, that is, food, but the peak availability might occur at drastically different times when compared to the natural environment.

The wild boar as our model host species is a long‐lived mammal with seasonal breeding that has an annual peak and is currently profiting from climate warming‐induced changes of the environment (Vetter et al., 2020). Pathogens will most probably profit most in species with multiple annual peaks of reproduction. Multiple reproductive events per year, like in hyenas (Kruuk, 1972), might mitigate effects of a mismatch on host–pathogen coexistence. While during one peak the resources might be scarce and the population size would be temporally reduced, the time between several birth peaks could be short enough to compensate the drop in host density and benefit host–pathogen coexistence. Subsequently, the more birth peaks a species has, the less it should be affected by temporal mismatches. In case of wild boar, if the species continues to benefit from the effects of climate and land‐use change, the single birth peak dynamic might continue to change toward multiple reproductive peaks per year, further offsetting adverse effects on host–pathogen coexistence. This could lead to an upsurge in persistent viral outbreaks throughout wild boar populations, which might exacerbate currently discussed processes like individual infection risk in piglets and movement (Scherer et al., ,^2019^, ^2020^). A prominent example is the persistence of African Swine Fever (ASF) in wild boar in Europe, which affects animal health more severely and can cause profound economic damages (Halasa et al., 2016) when coming into contact with domestic pigs.

Additionally, due to high mutation rates and short generation times, pathogens are likely to evolve, which can influence host as well as pathogen survival (Galvani, 2003) and might compensate for the response of the host species to changes in resource availability. Seasonal resource dynamics might strongly affect pathogen evolution if, during periods of high host densities, a particular strain of the pathogen has adapted to capitalize on the increased possibility for transmission or during periods of low host density if the pathogen has adapted to persist through those conditions (Altizer et al., 2006; Hite & Cressler, 2018; Koelle et al., 2005). A strong temporal mismatch that creates disease hotspots in combination with an even stronger system of alternating high and low host population densities than basic seasonality could further facilitate pathogen evolution. Furthermore, our model does not account for the host immune system, which can be impacted by a dynamic resource landscape. Long periods of resource scarcity and an expected poorer nutrition, as well as increased investment in movement to move toward higher resource areas, have been shown to negatively affect a host individual's ability to defend against infectious diseases (Altizer et al., 2006; Sheldon & Verhulst, 1996; White et al., 2018b). Subsequently, this could lead to further alterations of host–pathogen dynamics and coexistence.

In conclusion, our work has shown that temporary spatial hotspots of infectious hosts can emerge from a limited number of high‐resource sites that are formed due to temporal mismatches between resource availability and host reproduction. Considering the increasing effect of climate and land‐use change on resource availability and distribution, this will promote the understanding of how temporal resource variability and host movement affect host–pathogen systems.

### 4.1 Statement of authorship

All authors agree to submission of the manuscript, and each author carries a degree of responsibility for the accuracy, integrity, and ethics of the manuscript and works described therein.

## CONFLICT OF INTEREST

The authors declare no conflict of interest.

## AUTHOR CONTRIBUTION

**Tobias Kürschner:** Conceptualization (equal); Formal analysis (equal); Methodology (equal); Writing‐original draft (lead); Writing‐review & editing (equal). **Cédric Scherer:** Formal analysis (equal); Methodology (equal); Writing‐review & editing (equal). **Viktoriia Radchuk:** Formal analysis (equal); Writing‐review & editing (equal). **Niels Blaum:** Writing‐review & editing (equal). **Stephanie Kramer‐Schadt:** Conceptualization (equal); Formal analysis (equal); Methodology (equal); Writing‐review & editing (equal).

## Supporting information

Supplementary Material

## Data Availability

The model implementation in NetLogo is available on Zenodo (https://doi.org/10.5281/zenodo.4593791).
