# Supplementary material for: Movement can mediate temporal mismatches between resource availability and biological events in host–pathogen interactions
Source: Ecol Evol. 2021 Mar 29;11(10):5728–41. doi: 10.1002/ece3.7478 (PMC8131764; doi:10.1002/ece3.7478)
Supplement: Supplementary file 1 — Supplementary Material [file ECE3-11-5728-s001.pdf]

Supplementary material

Content:

- Appendix S1: Model description (ODD protocol)
- Appendix S2: Additional figures

Appendix S1: Model description (ODD protocol)

The classical swine fever (CSF) wild boar model is a combination of a spatially explicit, stochastic, agent-based model for wild boars (*Sus scrofa* L.) and an epidemiological model for the CSF virus. The model was implemented in NetLogo (Wilensky, 1999) and is documented following the ODD protocol (Grimm et al., 2006, 2010, 2020).

The ODD protocol below includes changes in model description across two wild boar / CSF eco-epidemiological models. The first model SwiFCoIBMove (M1 and black text throughout, Scherer et al. 2019, 2020) was developed to assess the effects of host movement and landscape structure on disease dynamics and the one described here, SwiFCoIBM_dynamic (M2 and blue text throughout) was developed to assess the role of a dynamic resource landscape and temporal mismatches on disease dynamics. As a note, while in some instance (as seen for example in 1. Purpose) parts of the original model are replaced, in other instances and submodels (as seen in 4b. Emergence) M2 is a mixture of old and new versions.

1. Purpose

[1]M1: The model aims to assess the role of host movement based on individual movement decisions (imposed, i.e. a correlated random walk, or based on external factors such as habitat and conspecifics) and landscape structure in driving on-going disease dynamics as pathogen persistence and patterns of spread. Specifically, the question will be addressed to what degree a high-resolution representation of the interaction between moving vectors and landscape features is required to capture spatio-temporal disease dynamics in a predictive way.

M2: The model aims to assess the role of a dynamic resource landscape and temporal shifts in resources on disease dynamics as pathogen persistence and patterns of spread. Specifically, how seasonal resource availability and the decoupling of resource availability and reproduction alter disease dynamics.

2. Entities, state variables and scales

The model entities are spatial units, or grid cells, and wild boars. Grid cells are characterized by habitat quality in terms of breeding capacity, i.e. the number of female boars that are allowed to have offspring (Jedrzejewska, Jedrzejewski, Bunevich, Milkowski, & Krasinski, 1997). Thereby, local host density is in the model, i.e. increasing numbers of fertile females can breed only until breeding capacity is reached. A grid

[1]This ODD describes M2, so that 'Purpose' for M1 is obsolete. It is kept in the ODD to better understand how the design of the model developed

cell represents about 2 km × 2 km, encompassing an average home range area of a boar group (Leaper, Massei, Gorman, & Aspinall, 1999).

Wild boars are characterized by sex, age in weeks, location, demographic status (e.g. breeding, dispersing and ranging) and health status. Three age classes are distinguished: piglet (< 34 weeks), subadult (between 34 weeks and < 1 year for females and < 2 years for males) and adult. Location is defined by the grid cell the wild boar inhabits. The health status of the individuals is described by an SIR epidemiological classification (susceptible; transiently infected; lethally infected with individual infectious period; immune by surviving the infection or by maternal antibodies). Females, which are at least subadult, may be assigned as breeders according to the breeding capacity of their family group's cell. Subadult wild boars may disperse during the dispersal period dependent on their sex and demographic status (disperser or non-disperser). In the model versions simulating explicit movement, males become ranging individuals (i.e. move solely) once they are adult and thereby change their locations every time step.

M1 & M2: One time step corresponds to the approximate CSF incubation time of one week (Artois et al., 2002; Moennig, Floegel-Niesmann, & Greiser-Wilke, 2003) while simulations usually run for 12 years (624 weeks) or 50 years (2600 weeks) with the virus being released in the second year (week 53–104) to a defined boar group to ensure the same distance to the model borders and an established spatial population structure. The model landscape consists of 100 km × 50 km (50 × 25 grid cells).

3. Process overview and scheduling

[1]M1: Each time step, the following procedures are executed by the wild boars in the given order: pathogen transmission, ranging movement, natal dispersal of males and females, respectively, reproduction, mortality, ageing, and disease course (Fig. S1)

In the first week of each year, females are assigned to breed. Natal dispersal of males and females was limited to week 17 and week 29 of the year, respectively.

M2: Each time step, the following procedures are executed by the wild boars in the given order: pathogen transmission, ranging movement, natal dispersal of males and females, resource-based host movement, host reproduction, general host mortality, resource-based host mortality, ageing, and disease course (Fig. S1.1).

[1] This ODD describes M2, so that 'Process overview and scheduling' for M1 is obsolete. It is kept in the ODD to better understand how the design of the model developed

M2: Females are assigned to breed on a week to week basis, with the prerequisite that they have not bred previously in the current year.

4. Design concepts

a) Basic principles

M1 & M2: Both processes of the host and the pathogen are simulated with a given stochasticity which resembles natural conditions. Disease transmission for groups is modelled density-dependent (see Eq. 1 and 2). Heterogeneous landscape scenarios were simulated using neutral landscape model algorithms.

b) Emergence

M1 & M2: Wild boar population dynamics emerge from individual behaviour, resulting from age- and sex-dependent movement behaviours (natal dispersal and ranging) as well as age-dependent seasonal reproduction, survival probabilities and is mitigated by the underlying dynamic resource landscape. The epidemic course emerges from different virus transmission probabilities, wild boar movement behaviours as well as individual stochastic disease courses and infectious periods.

c) Adaptation

M1 & M2: Movement rules either reproduce observed movement behaviours (phenomenological approach, e.g. correlated random walk with a given directional persistence) or are based on decisions in response to spatial structure or conspecifics (mechanistic approach, e.g. the competition-driven movement rule implicitly seeks to increase habitat quality in relation to conspecifics). The number of breeding females per cell is determined by (a) the habitat quality of the cell and M1: (b) the number and age of the reproductive females within the cell.

d) Objectives

M1 & M2: The exact way individuals decide where to move depend on the applied movement rule (see section “Ranging movement”). In general, ranging individuals (i.e. adult males) using decision-based movement rules move into the neighbouring cell with the highest weight (which is defined by the applied movement rule). Individuals’ decisions are not perfect but may be a random direction depending on the directional persistence ρ (probability to move randomly = $1 - \rho$, set to 0.7).

Dispersing individuals search for empty (unoccupied) cells (in case of female dispersers) or join other groups that are below their cells’ carrying capacity (in case of male dispersers).

e) Learning

M1 & M2: There is no learning implemented in our model.

f) Prediction

M1 & M2: There is no need to predict future environmental conditions.

g) Sensing

M1 & M2: Individuals moving through the landscape may base their decisions on the underlying landscape structure in terms of habitat quality (or, synonymously, breeding capacity *B*) or number of conspecifics. Moving individuals can sense all that information from their own cell and surrounding cells but the decision might be random (implicitly due to insufficient information) depending on the assumed directional persistence ρ.

h) Interaction

M1 & M2: Based on the transmission rates, interactions between individuals might lead to virus transmission from an infected to a susceptible individual. Reproduction is density-and age-dependent with the oldest females giving birth first. Thus, if the number of reproducible females exceeds a cell's breeding capacity, interaction leads to repressed reproduction.

i) Stochasticity

M1 & M2: Demographic and behavioural parameters are imposed via probability distributions to account for variation in the biological processes. Stochastic individual disease courses and infectious periods are modelled explicitly because variation in the disease outcome between individuals was identified to be essential for virus endemicity without reservoirs (Kramer-Schadt, Fernández, Eisinger, Grimm, & Thulke, 2009). Movement decisions are based on a random component (with 30% of the decisions being random).

j) Collectives

M1 & M2: Individuals that are not ranging between home ranges (i.e. all individuals but adult males) form groups within their cell that experience density-dependent transmission probabilities. These groups are formed by reproducible females and their offspring. During natal dispersal, male subadults may join those groups while female subadults may form new groups.

k) Observation

M1 & M2: To evaluate model outcomes, we measure several properties of the host-virus system at each time step of the simulation. These outputs include number of individuals for each class of age (piglet,

subadult, adult), demographic (resident, ranging) and health status (susceptible, infected, recovered) and their combinations. Furthermore, sex ratio, number of new infections (transient and lethal), number of cells with infectious individuals, the last week of infection and the cumulative time (i.e. how many weeks in total) of infected hosts per habitat cell are recorded. From this output, duration of an outbreak, probability of disease persistence (i.e. the virus being present in the system until the end of simulations), outbreak size for different classes, as well as density distribution of infected hosts can be estimated.

5. Initialization

M1 & M2: We simulate landscapes with four different levels of spatial heterogeneity in habitat quality: homogeneous (only M1), random, and two or four clustered landscape scenarios using an random cluster algorithm (very small, small, medium and large clusters, respectively; Saura & Martínez-Millán, 2000; Table S1 and S1.1) generated using the R package NLMR (Sciaini, Fritsch, Scherer, & Simpkins, 2018). In homogeneous landscapes, one boar family group was allocated to each cell with an average breeding capacity *B* of 4.5 females, resulting in the reported density of approximately 20 boars per cell or 5 boars per km^2 (EFSA (European Food Safety Authority), 2009; Howells & Edwards-Jones, 1997; Melis, Szafranska, Jedrzejewska, & Barton, 2006; Sodeikat & Pohlmeyer, 2003). In heterogeneous landscapes a breeding capacity *B* between 0 and 9 was assigned to each of the 2500 grid cells ($B \in \{0;9\}$). In case *B* is a floating point number, the number of individuals is determined stochastically based on the remainder. Initial age distribution was obtained from the results of a 100-year model run conducted by (Kramer-Schadt et al., 2009); Table S2), the sex ratio was balanced (i.e. probability of 0.5 to be either male or female). Wild boar density reflects long-term average values of densely populated Central European habitats (EFSA (European Food Safety Authority), 2009; Howells & Edwards-Jones, 1997; Melis et al., 2006; Sodeikat & Pohlmeyer, 2003). Group size was initialised according to the cells carrying capacity K_i that is 4.5 times its breeding capacity B_i.

6. Input

M1 & M2: The model does not include external input representing environmental conditions changing over time.

7. Submodels

7.1. Initial infection (pathogen release)

M1 & M2: The virus is released to the population by infection of one wild boar group (i.e. cell) in middle of the upper row of home ranges (i.e. cell with the coordinates x = 1 and y = 13) to allow for comparison between runs. The release is scheduled in a random week of the second year of the model run.

7.2. Pathogen transmission

M1 & M2: Virus transmission is modelled stochastically. The transmission parameter determines the weekly probability of being infected by an infectious group mate β_w.

In the model without movement (afterwards referred to as the "classical model"), weekly infection pressure λ_i for each susceptible individual in cell i is determined by the probability of being infected by an infectious group mate β_w (within-group transmission probability) and the probability of being infected by an infectious individual in one of the eight neighbouring cells β_e (between-group transmission probability):

$$\lambda_i = 1 - (1 - \beta_w)^{I_i} \cdot (1 - \beta_e)^{\Sigma_i I_j} \quad [1]$$

where I_i is the number of infectious group mates and I_j is the numbers of infectious hosts in the jth adjacent cell. The resulting probability value λ_i provides the parameter of a binomial chance process to decide whether a susceptible animal will be infected.

The transmission parameters β_w (and thereby β_e since it was fixed as one tenth of β_w) was calibrated in order to reproduce the spreading velocity observed in France (Rossi et al., 2010) with the constant parameter value β_w = 0.0208 within and, hence, β_e = 0.00208 between groups (Kramer-Schadt et al., 2009).

In the model version involving explicit movements presented here, infection might be translocated within the host population. Because ranging movements are performed by adult males only, the homogeneous transmission probability λ_i was transformed to account for these sex-and age-dependent transmission probabilities. Therefore, for all individuals but adult males the weekly probability of being infected by infectious group mates (but not by infectious ranging males) is simply β_w since females and their group follow a staying rule with the almost all contacts within the group (Pepin et al., 2016; Spitz & Janeau, 1990). The accumulated probability λ_i for susceptible non-ranging individuals is thus reduced to:

$$\lambda_i = 1 - (1 - \beta_w)^{I_i} \quad [2]$$

where is the number of infectious group mates (but not infectious ranging males which do not belong to any group). For adult, ranging males, the individual probability of virus transmission during movements β_m (i.e. being infected by an infectious animal in a cell moving through as well as the probability of infecting a susceptible individual while passing the cell) was fitted to account for the resuming transmission probabilities (i.e. the remaining within- and between-group transmission probabilities of the classical model, see Eq. 2). We calibrated one constant transmission probability (i.e. $\beta_w = \beta_m$, afterwards just referred to as simply β) to result in the comparable distributions of the basic reproduction number (R_0, number of secondary infections) as in the classical model. Using a Kolmogorov-Smirnov test, the constant

parameter value β = 0.022 gave the best results for different scenarios of infection and movement rules (for details see Appendix S2). Individual per step transmission probability $\beta_{m,i}$ is related to the number of steps the animal makes. Therefore, the infection probability for cells passed by infected individuals is scaled to the individual movement distance $d_{m,i}$ (see section "Ranging movement"):

$$\beta_{m,i} = \frac{\beta}{d_{m,i}}. \quad [3]$$

By doing so, infection probability decreases with increasing movement distance which relates to the time an animal is able to spend within each cell and thus to transmit the virus on its way.

CSF shows a variety of disease courses on the individual level (Depner et al., 1997; Liess, 1987). Therefore, in our model the disease course is stochastically specified for each individual. The disease course submodel is described by two parameters: individual case mortality M and the mean infectious period of lethally infected hosts μ. Upon infection the host is stochastically assigned either as lethally infected (with probability *M*) or as transiently infected (1 – *M*). *M* is age-specific (Dahle & Liess 1992): for adults the probability is decreased to $M_a = M^2$ and for piglets increased to $M_p = \sqrt{M}$ while it is unchanged for subadults $M_s = M$. Transiently infected wild boars first pass through an infectious period of one week and subsequently becomes non-infectious and gain life-long immunity (Artois et al. 2002; Moennig et al. 2003; EFSA 2009). The individual infectious period (m_i in weeks) of lethally infected hosts is drawn from an exponential distribution with the mean specified by parameter μ:

$$m_i = 1 + floor(-(\mu - 0.5) \cdot ln(U(0,1))) \quad [4]$$

where *U(0,1)* is a uniformly distributed random number between 0 and 1. To avoid unrealistically long infections, m_i was stochastically assigned until $m_i \leq 10 \times \mu$. Lethally infected hosts remain infectious until death. Offspring from immune female breeders gets maternal antibodies and is thus immune for the first eight to twelve weeks after birth (t_{anti}). The number of weeks of immunisation due to maternal antibodies is randomly assigned for those piglets.

7.3. Ranging movement

M1 & M2: While adult females move mainly within their family group (*staying strategy*), adult males follow a *ranging strategy* and move solitary (Spitz & Janeau 1990, Fig. 2). Ranging distances vary between males with a mean distance of 24 km per week and rare long-distance behaviour with up to 84 km per week (Morelle et al. 2015). For each adult male *i*, an individual movement distance $d_{m,i}$ was drawn from the median of the Weibull distribution given by *b*(1 - ln(1 - U[0,1]))^k* with a scale of *b* = 26 and a shape of *k* = 1.3, resulting in a mean of 12 cells and truncated to the maximum movement distance D_{max} of 42

cells (with a cell being 2 km in diameter). To study effects of inter-cell movement on disease dynamics, we implemented three different movement strategies. In general, ranging individuals move until the individual weekly movement distance is reached or there is no better decision to make and only into cells with a positive habitat quality (*B* > 0). Individuals stop moving if there is no cell available. If there is a tie (i.e. two or more cells with equal attractiveness), one of those cells is chosen randomly.

M1 & M2: *(a) Correlated Random Walk (CRW):* In a CRW, subsequent movement directions are correlated such that highly correlated movement paths are nearly straight (Turchin 1998; Fig. S3a). This movement model improves the RW approach by incorporating directional persistence that moving animals very frequently exhibit (Fig. S3; Kareiva & Shigesada 1983). The direction of a step is drawn from a wrapped Cauchy distribution with a mean direction equal to the previous direction (Fletcher 2006):

$$\theta_{t,i} = \theta_{t-1,i} + 2 \cdot arctan\left(\frac{1-\rho}{1+\rho} \cdot \pi \cdot U(-0.5{,}0.5)\right) \qquad [5]$$

Where $\theta_{t,i}$ and $\theta_{t-1,i}$ are the headings of individual *i* of the next or previous step, respectively, ρ is a parameter related to the concentration around the mean direction (i.e. $\rho = 1$ results in a straight line and $\rho = 0$ equals a RW), and U(–0.5,0.5) is a uniformly distributed random number between –0.5 and 0.5. In case individuals reach the border of the simulated landscape, the Individual is reflected in a 90° angle followed by reset and recalculation of $\theta_{t,I}$ to avoid aggregation around the landscape borders.

M1 & M2: *(b) Habitat-Dependent Movement (HDM):* A reasonable hypothesis is that a selective animal should choose high quality cells for its home range. The distribution and abundance of resources greatly affect movement patterns of several animal species and has also been reported for wild boars (Morelle et al. 2015). Assuming higher food resources and better shelter in high quality habitat, individuals decide among their neighbouring cells based on the habitat quality (Fig. S3b). The parameter ρ here relates to the accuracy of an individual estimating the quality of the focal and neighbouring cells, with the movement decision in ρ–1 cases being purely random.

[1]M1: *(c) Competition-Driven Movement (CDM):* Effective habitat quality might be lower due to competition with conspecifics. Thus, competition-driven movement is biased to cells with higher net habitat quality, and was implemented as negative density dependence. Movement weights were calculated as the ratio of number of conspecifics in the focal cell and the carrying capacity (*K*), i.e. the potential maximum group size of the cell (Fig. S3c).

[1] This ODD describes M2, so that 'Competition-Driven Movement (CDM)' for M1 is obsolete. It is kept in the ODD to better understand how the design of the model developed

For all movement strategies, ρ was fixed as 0.7, i.e. 30% of the decisions were made randomly.

7.4. Natal dispersal

M1 & M2: Herd splitting, where subadult individuals may move together to search for or form new groups, is performed in specified weeks of the. The timing of these events is sex-dependent: Subadult females without offspring perform their natal dispersal in the 29th week of the year, while subadult males disperse during the 17th week. In the given week of the year, all herds to split are extracted, matching the conditions of containing at least a specified number of subadults N_{disp} to move (either male or female). For female dispersal, only cells exceeding the breeding capacity *B* are evaluated. For each of them, a habitat cell not exceeding carrying capacity *K* (for males) or without any family group (for females) within a Euclidean distance d_{disp} is selected randomly as new cell for the group, excluding the source cell (Fig. 2). If there is no cell fulfilling these conditions, subadults stay within their group's cell.

7.5. Reproduction

M1 & M2: Females reproduce once a year, depending on their age class. Individual females, which are at least subadult, reproduce depending on the season with a peak in March and no reproduction in winter from October to December (Boitani et al. 1995) (Table S2).

M1 & M2: In the first week of the year, female individuals are checked for their breeding status; female individuals are checked for their breeding status on a weekly basis. All females not exceeding their habitat cells breeding capacity, starting with the oldest individuals, are allowed to breed.

M1: The week of the year to breed is assigned in the first week of each year according to weekly reproduction probabilities, derived from monthly probabilities and the number of weeks in the month (Table S2).

M2: If an individual was assigned to breed the chance of reproduction at that time is drawn from monthly probabilities and the number of weeks in the month (Table S2).

M1 & M2: Litter size is drawn from a pre-calculated truncated normal distribution (Table S1,3) and reduced to a constant fraction for infected individuals. Litter size of transient shedders and lethally infected hosts is multiplied with the reduction factor α_f.

M1 & M2: Depending on the disease state of the breeding individual, its piglet's disease states are adjusted. Susceptible individuals produce susceptible offspring, immune individuals produce immune offspring with maternal antibodies (see section "Pathogen state transition"). Transient shedders and

lethally infected individuals yield offspring, each one lethally infected with a given probability of prenatal infection p_{pi}.

7.6. Mortality

M1 & M2: Stochastic baseline mortality is age-dependent and adjusted to annual survival estimates found in the literature (Table S1). Per time step we apply the adjusted age-dependent mortality (m_{week}) to the individual:

$$m_{week} = 1 - (s_{year})^{1/52}. \quad [6]$$

In addition to the stochastic baseline mortality, each individual may die due to reaching a certain maximum age, or due to a lethal infection after a certain infection time span m (see section "Pathogen transmission").

M2: A selected number of individuals K_{over} (see 7.10 Resource response for the selection) are subject to a variable, resource-based mortality with an increasing chance of mortality dependent on how long the individual is subject to low resource conditions up to a variable time limit S_{max} as well as the number of individuals (g_{act}) in relation to maximum group (g_{sus}) size and age.

$$m_{resource} = \frac{(g_{act} - g_{sus})}{100} * a * t_{over} \quad [7]$$

With a being an age factor corresponding to the individuals being a piglet or adult/subadult and t_{over} the time the individual is part of the K_{over} individual pool. As well as a 100% mortality when t_{over} equals S_{max}.

7.7. Ageing

M1 & M2: The ageing process iterates over all individuals. For each individual k, age T_k is incremented one week and disease state transitions are performed. Females become subadult and adult at an age of 34 and 52 weeks, respectively, while males enter the subadult and adult age groups at an age of 21 and 104 week, respectively.

7.8. Disease course (pathogen state transition)

M1 & M2: Transient shedders convert to immune after a certain latency period t_{latent}. An individual i protected by maternal antibodies turns susceptible if reaching an age T_i of the protection time of t_{anti} (see section "Pathogen transmission"). After disease state transition the age of the infection is incremented by one week if the individual is not susceptible.

7.9. Landscape dynamic

M2: The autocorrelated landscape dynamic is superimposed over the different types of heterogeneous starting landscapes. Throughout each simulated year, resource availability in each cell increases in 5-weeks intervals for approximately 25 weeks and then declines in 5-week intervals for the next 25 weeks. The resource availability translates directly into the breeding capacity for each cell and for each increase or decrease the value of the breeding capacity is changed by one. During the increase, the breeding capacity cannot exceed the maximum breeding capacity of 9 and cannot fall below 1 during the decrease i.e. the breeding capacity in a cell designated as habitat cannot become 0 during the course of a year. Additionally, barriers and matrix which have an assigned breeding capacity of 0 cannot become habitat i.e. matrix/barrier cells will always have a breeding capacity of 0. The default landscape dynamic is implemented to follow the host individual's monthly reproduction probabilities (see table S2), where the peak reproduction probability matches in time with the peak resource availability. Furthermore, the landscape dynamic can be temporally shifted away from the peak host reproduction probability in 25% increments (0% - match, 25% mismatch, 50% mismatch, 75% mismatch, 100% - full mismatch). In this case, a full mismatch would mean that the resource availability is at its lowest when the host reproduction probability is at its highest.

The random landscape dynamic functions similar to the autocorrelated variant without a temporal shift. Throughout each simulated year, resource availability in each cell changes randomly in 5-weeks intervals for approximately 50 weeks whereby each cell is allocated a random integer between 1 and 9. At the end of each year, the landscape resets to the original starting landscape.

7.10. Resource responses

M2: If the group size exceeds the carrying capacity *K* in a cell, a dispersal event will be triggered. The likelihood of dispersal follows an age gradient where older females will try to disperse last. For male individuals, a random habitat cell not exceeding the carrying capacity *K* will be randomly selected within a Euclidean distance d_{disp} as new cell. For female individuals, the randomly selected habitat cell must fulfil the prerequisite of not hosting another family group. However, if an individual currently has offspring it and the offspring will stay at in their current cell. If there is no suitable habitat cell fulfilling these prerequisites within the distance d_{disp} the individuals will stay. Individuals are continuously selected for dispersal until the group size no longer exceeds the carrying capacity *K* or no more suitable habitat within the distance d_{disp} can be found. For a secondary response after the dispersal event (if applicable), a cell

specific number of individuals K_{over} that is the difference between carrying capacity K and group size will be selected. The selection of these individuals is age dependant where the oldest females are selected last. All selected individuals are subjected to an increasing age-dependant mortality (see mortality).

References

Artois, M., Depner, K. R., Guberti, V., Hars, J., Rossi, S., & Rutili, D. (2002). Classical swine fever (hog cholera) in wild boar in Europe. *Revue Scientifique et Technique* , *21*(2), 287–303. Retrieved from https://www.ncbi.nlm.nih.gov/pubmed/11974616

Bieber, C., & Ruf, T. (2005). Population dynamics in wild boar *Sus scrofa*: ecology, elasticity of growth rate and implications for the management of pulsed resource consumers. *The Journal of Applied Ecology*, *42*(6), 1203–1213. doi: 10.1111/j.1365-2664.2005.01094.x

Boitani, L., Trapanese, P., Mattei, L., & Nonis, D. (1995). Demography of a wild boar (*Sus scrofa*, L.) population in Tuscany, Italy. *Gibier, Faune Sauvage / ONC, Office National de La Chasse*, *12*(2), 109–132.

Depner, K. R., Hinrichs, U., Bickhardt, K., Greiser-Wilke, I., Pohlenz, J., Moennig, V., & Liess, B. (1997). Influence of breed-related factors on the course of classical swine fever virus infection. *The Veterinary Record*, *140*(19), 506–507. Retrieved from https://www.ncbi.nlm.nih.gov/pubmed/9172299

Depner, K. R., Müller, T., Lange, E., Staubach, C., & Teuffert, J. (2000). Transient classical swine fever virus infection in wild boar piglets partially protected by maternal antibodies. *Deutsche Tierarztliche Wochenschrift*, *107*(2), 66–68. Retrieved from https://www.ncbi.nlm.nih.gov/pubmed/10743336

Durio, P., Gallo Orsi, U., Macchi, E., & Perrone, A. (2014). Structure and monthly birth distribution of a wild boar population living in mountainous environment. *Journal of Mountain Ecology*, *3*(0). Retrieved from http://www.mountainecology.org/index.php/me/article/view/112

EFSA (European Food Safety Authority). (2009). Control and eradication of Classical Swine Fever in wild boar. *European Food Safety Authority Journal*, *932*(EFSA-Q-2007-200), 1–18. Retrieved from https://efsa.onlinelibrary.wiley.com/doi/abs/10.2903/j.efsa.2009.932

Fernández-Llario, P., Carranza, J., & Mateos-Quesada, P. (1999). Sex allocation in a polygynous mammal with large litters: the wild boar. *Animal Behaviour*, *58*(5), 1079–1084. doi: 10.1006/anbe.1999.1234

Fletcher, R. J., Jr. (2006). Emergent properties of conspecific attraction in fragmented landscapes. *The American Naturalist*, *168*(2), 207–219. doi: 10.1086/505764

Focardi, S., Toso, S., & Pecchioli, E. (1996). The population modelling of fallow deer and wild boar in a Mediterranean ecosystem. *Forest Ecology and Management*, *88*(1), 7–14. doi: 10.1016/S0378-1127(96)03804-2

Gaillard, J. M., Vassant, J., & Klein, F. (1987). Quelques caractéristiques de la dynamique des populations de sangliers (*Sus scrofa scrofa*) en milieu chassé. *Gibier, Faune Sauvage / ONC, Office National de La Chasse*, *4*, 31–47.

Grimm, V., Berger, U., Bastiansen, F., Eliassen, S., Ginot, V., Giske, J., … DeAngelis, D. L. (2006). A standard protocol for describing individual-based and agent-based models. *Ecological Modelling*, *198*(1), 115–126. doi: 10.1016/j.ecolmodel.2006.04.023

Grimm, V., Berger, U., DeAngelis, D. L., Polhill, J. G., Giske, J., & Railsback, S. F. (2010). The ODD protocol: A review and first update. *Ecological Modelling*, *221*(23), 2760–2768. doi: 10.1016/j.ecolmodel.2010.08.019

Grimm, V. et al. 2020. The ODD Protocol for Describing Agent-Based and Other Simulation Models: A Second Update to Improve Clarity, Replication, and Structural Realism. - JASSS 23: 7.

Howells, O., & Edwards-Jones, G. (1997). A feasibility study of reintroducing wild boar *Sus scrofa* to Scotland: Are existing woodlands large

enough to support minimum viable populations. *Biological Conservation*, *81*(1), 77–89. doi: 10.1016/S0006-3207(96)00134-6

Jedrzejewska, B., Jedrzejewski, W., Bunevich, A. N., Milkowski, L., & Krasinski, Z. A. (1997). Factors shaping population densities and increase rates of ungulates in Bialowieza Primeval Forest (Poland and Belarus) in the 19th and 20th centuries. *Acta Theriologica*, *4*(42). Retrieved from https://www.infona.pl/resource/bwmeta1.element.agro-article-f12b76e6-383a-4497-809a-9ca2f1205a93

Jezierski, W. (1977). Longevity and mortality rate in a population of wild boar. *Acta Theriologica*, *22*(24), 337–348. Retrieved from http://rcin.org.pl/dlibra/docmetadata?id=10471

Kramer-Schadt, S., Fernández, N., Eisinger, D., Grimm, V., & Thulke, H.-H. (2009). Individual variations in infectiousness explain long-term disease persistence in wildlife populations. *Oikos* , *118*(2), 199–208. Retrieved from https://onlinelibrary.wiley.com/doi/abs/10.1111/j.1600-0706.2008.16582.x

Leaper, R., Massei, G., Gorman, M. L., & Aspinall, R. (1999). The feasibility of reintroducing Wild Boar (*Sus scrofa*) to Scotland. *Mammal Review*, *29*(4), 239–258. doi: 10.1046/j.1365-2907.1999.2940239.x

Liess, B. (1987). Pathogenesis and epidemiology of hog cholera. *Annales de Recherches Veterinaires. Annals of Veterinary Research*, *18*(2), 139–145. Retrieved from https://www.ncbi.nlm.nih.gov/pubmed/3304111

Melis, C., Szafranska, P. A., Jedrzejewska, B., & Barton, K. (2006). Biogeographical variation in the population density of wild boar (*Sus scrofa*) in western Eurasia. *Journal of Biogeography*, *33*(5), 803–811. doi: 10.1111/j.1365-2699.2006.01434.x

Moennig, V., Floegel-Niesmann, G., & Greiser-Wilke, I. (2003). Clinical signs and epidemiology of classical swine fever: a review of new knowledge. *Veterinary Journal* , *165*(1), 11–20. Retrieved from https://www.ncbi.nlm.nih.gov/pubmed/12618065

Morelle, K., Podgórski, T., Prévot, C., Keuling, O., Lehaire, F., & Lejeune, P. (2015). Towards understanding wild boar *Sus scrofa* movement: a synthetic movement ecology approach. *Mammal Review*, *45*(1), 15–29. Retrieved from https://onlinelibrary.wiley.com/doi/abs/10.1111/mam.12028

Moretti, M. (2014). Birth distribution, structure and dynamics of a hunted mountain population of Wild boars (*Sus scrofa* L.), Ticino, Switzerland. *Journal of Mountain Ecology*, *3*(0). Retrieved from http://mountainecology.org/index.php/me/article/view/110

Pepin, K. M., Davis, A. J., Beasley, J., Boughton, R., Campbell, T., Cooper, S. M., … VerCauteren, K. C. (2016). Contact heterogeneities in feral swine: implications for disease management and future research. *Ecosphere* , *7*(3), e01230. doi: 10.1002/ecs2.1230

Rossi, S., Pol, F., Forot, B., Masse-Provin, N., Rigaux, S., Bronner, A., & Le Potier, M.-F. (2010). Preventive vaccination contributes to control classical swine fever in wild boar (*Sus scrofa sp.*). *Veterinary Microbiology*, *142*(1-2), 99–107. doi: 10.1016/j.vetmic.2009.09.050

Saura, S., & Martínez-Millán, J. (2000). Landscape patterns simulation with a modified random clusters method. *Landscape Ecology*, *15*(7), 661–678. doi: 10.1023/A:1008107902848

Scherer, C. et al. 2019. Seasonal host life-history processes fuel disease dynamics at different spatial scales (A Tate, Ed.). - J Anim Ecol 88: 1812–1824.

Scherer, C. et al. 2020. Moving infections: individual movement decisions drive disease persistence in spatially structured landscapes. - Oikos: oik.07002.

Sciaini, M., Fritsch, M., Scherer, C., & Simpkins, C. E. (2018). NLMR and landscapetools: An integrated environment for simulating and modifying neutral landscape models in R. *bioRxiv*. Retrieved from https://www.biorxiv.org/content/early/2018/04/27/307306.abstract

Sodeikat, G., & Pohlmeyer, K. (2003). Escape movements of family groups of wild boar *Sus scrofa* influenced by drive hunts in Lower Saxony, Germany. *Wildlife Biology*, *9*(1), 43–49. doi: 10.2981/wlb.2003.063

Spitz, F., & Janeau, G. (1990). Spatial strategies: an attempt to classify daily movements of wild boar. *Acta Theriologica*, *35*(1-2), 129–149.

Retrieved from http://rcin.org.pl/Content/11704/BI002_2613_Cz-40-2_Acta-T35-nr12-128-149_o.pdf

Wilensky, U. (1999). NetLogo (Version 6.0.4). Retrieved from http://ccl.northwestern.edu/netlogo/

Figures

Fig. S1: Flow chart of the SwiFCoIBMove model and its submodels

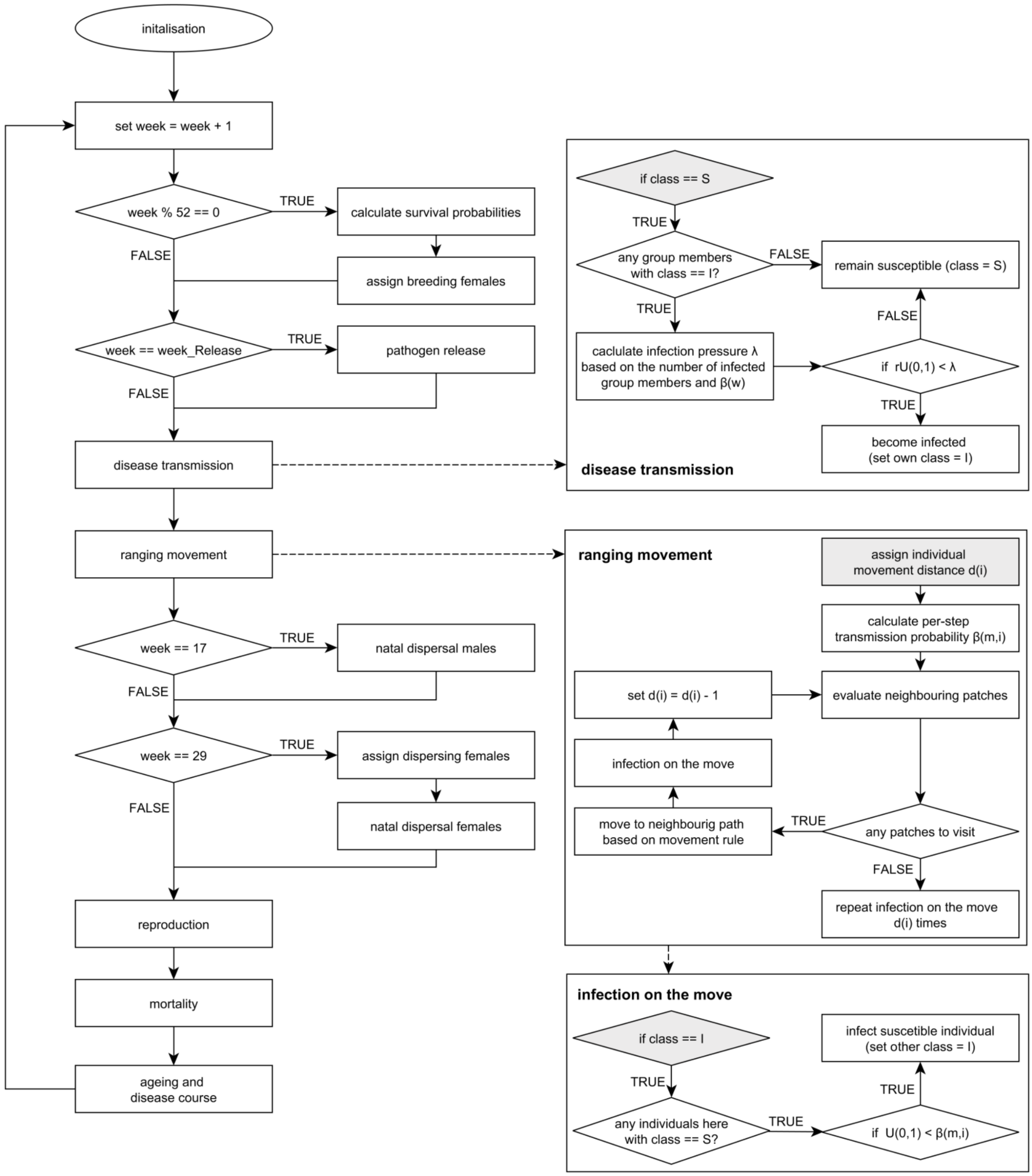

Fig. S1: Flow chart of the agent-based, spatially explicit host-pathogen model "SwiFCoIBMove" and its submodels.

Fig. S1.1: Flow chart of the SwiFCoIBM_dynamic model

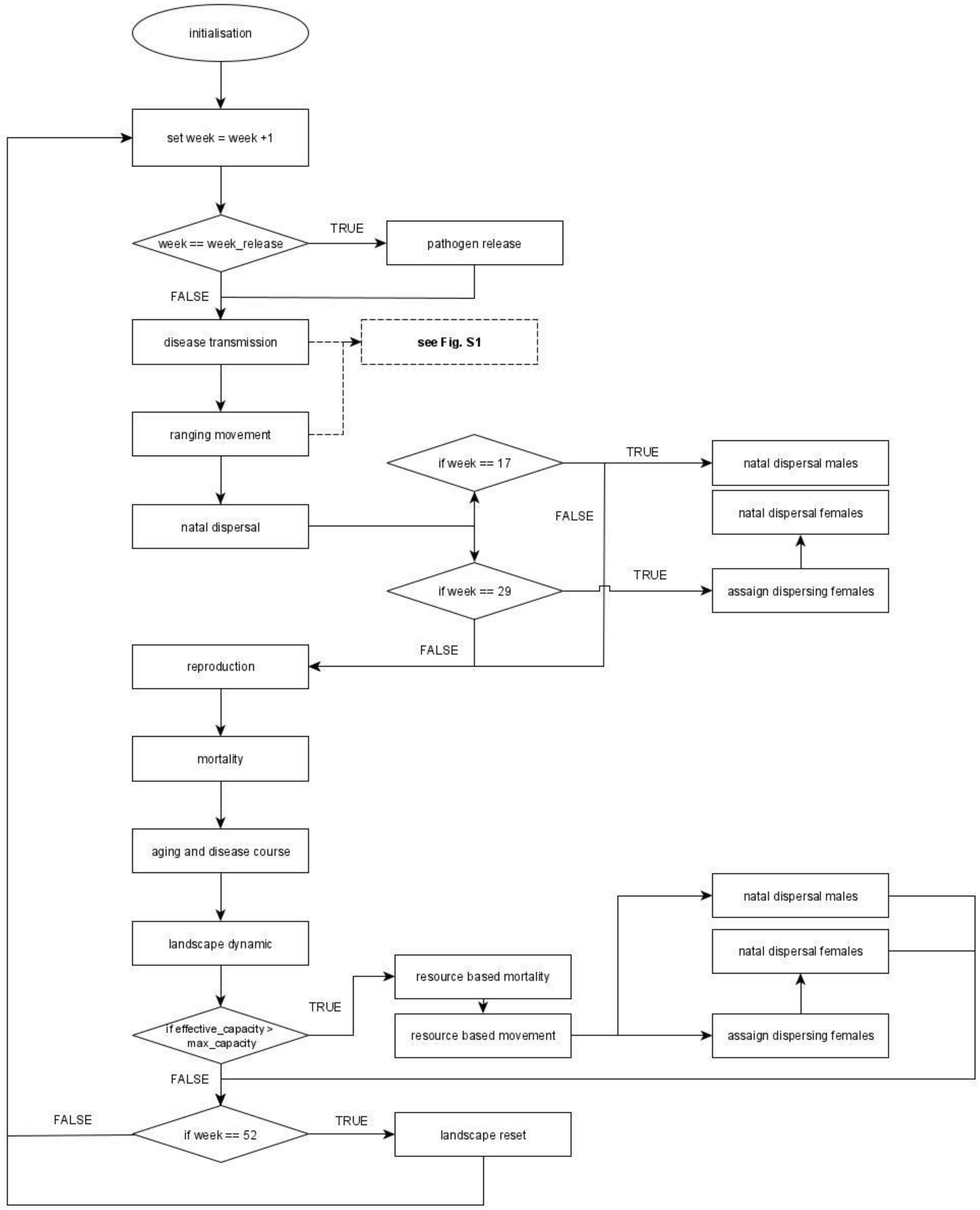

Fig. S1.1: Flow chart of the agent-based, spatially explicit host-pathogen model "SwiFCoIB_dynamic"

Fig. S2: Schematic overview of movement strategies in wild boar

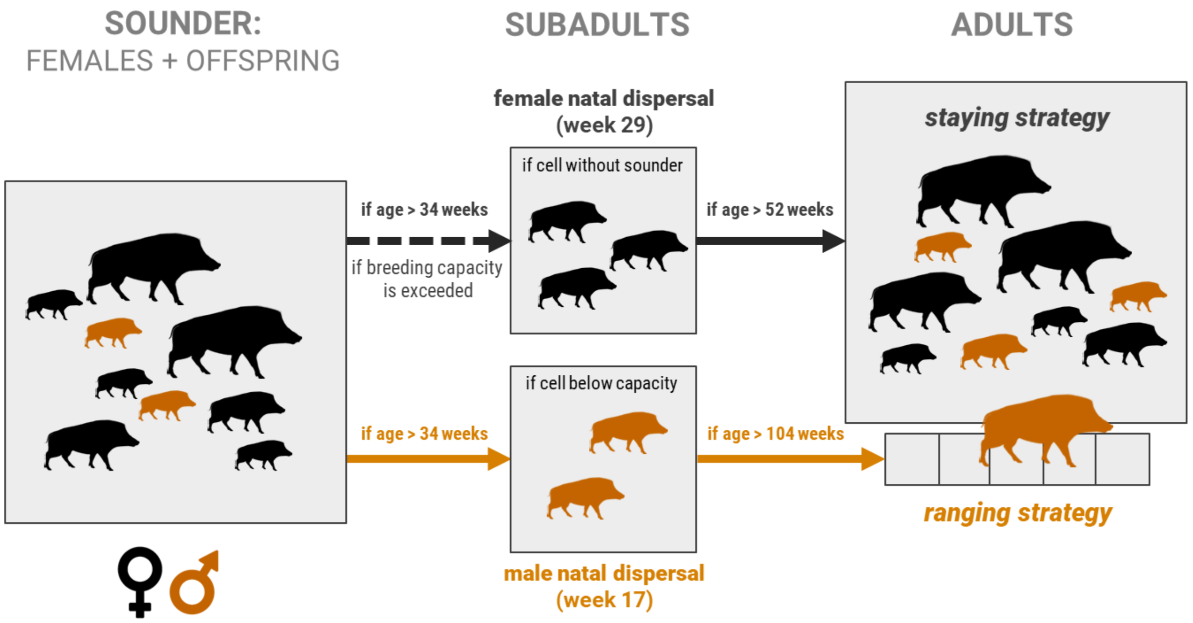

Fig. S2: Schematic representation of different natal dispersal events of subadult wild boars and movement strategies of adult wild boars. The group of adult and subadult females and their offspring is called a sounder. If the breeding capacity of the sounders cell is exceeded, subadult females search in groups of 3 or more individuals for an unoccupied cell in a radius of three cells. Female wild boars reach their adulthood earlier (model assumption: if older than 1 year) and follow a staying strategy, thus not moving outside of the sounders cell (grey squares). Male wild boars always separate from the sounder when reaching the subadult age and move in groups of 2 or more to one of the cells below capacity in a radius of 3 cells. When becoming adult, male wild boars move solitary between home ranges.

Fig. S3: Decision-making rules of the movement strategies

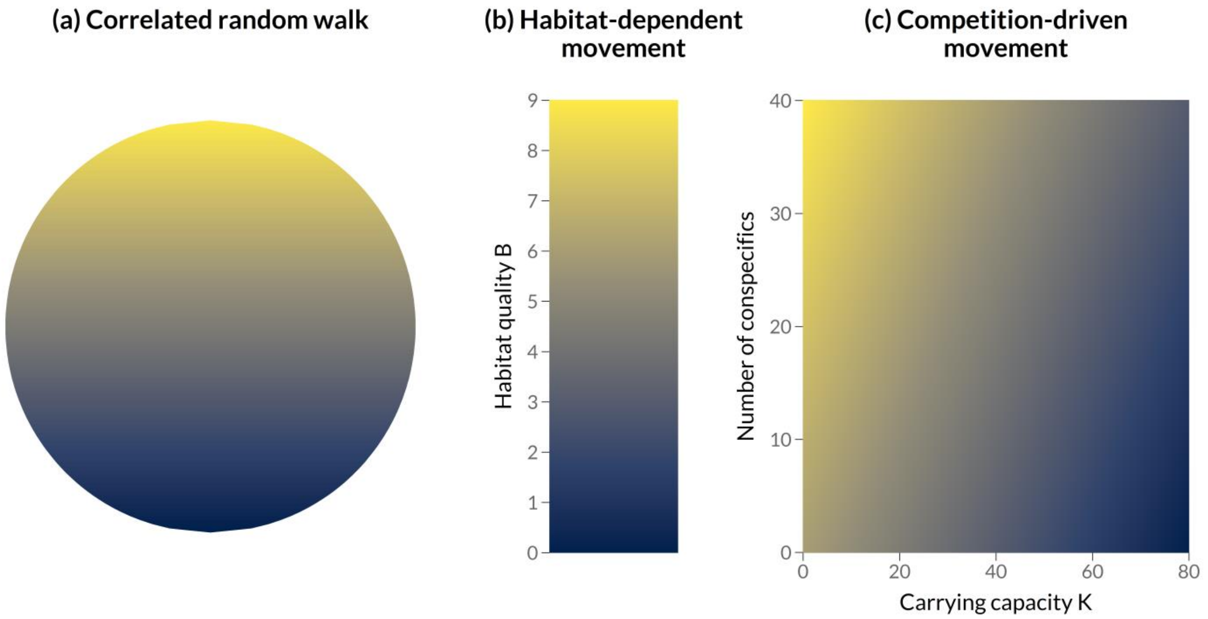

Figure S3: Movement assumptions used to model ranging behaviour of adult males. Probability of movement depends on the **(a)** direction of previous steps (Correlated Random Walk), **(b)** habitat quality *B* or **(c)** carrying capacity *K* in relation to the number of conspecifics. The lighter the colour, the higher the probability the individual chooses this cell. Movement decisions are furthermore driven by a random component determined by ρ.

Tables

Table S1: Parameter setup

Table S1: Parameter setup used in the spatially-explicit classical swine fever-wild boar model. M1: Each of the 60 combinations (3 movement strategies × 4 landscape scenarios × 5 case fatality ratios) was repeated 200 times while generating new underlying landscapes depending on the scenario input, resulting in 12.000 runs in total. M2: Each of the 300 combinations (3 movement strategies 3 × landscape dynamic × 4 landscape scenarios × 5 max survival time under low resources × 5 level of resource mismatch) was repeated 25 times, resulting in 22.500 runs in total.

**Parameter**	**Value M1**	**Value M2**	**Reference(s)**
Longevity	11 years (572 weeks)	11 years (572 weeks)	(Jezierski, 1977)
Sex ratio	1:1	1:1	(e.g. Durio, Gallo Orsi, Macchi, & Perrone, 2014; Fernández-Llario, Carranza, & Mateos-Quesada, 1999; Moretti, 2014)
Survival probability of adults and subadults	s_{mean}= 0.6; s_{min} = 0.4	s_{mean}= 0.6; s_{min} = 0.4	(Focardi, Toso, & Pecchioli, 1996; Gaillard, Vassant, & Klein, 1987)
Survival probability of piglets	s_{mean}= 0.5; s_{min} = 0.1	s_{mean}= 0.5; s_{min} = 0.1	(Focardi et al., 1996)
Reproduction probability	see Table S2	see Table S2	(Boitani, Trapanese, Mattei, & Nonis, 1995)
Breed count distribution	see Table S3	see Table S3	(Bieber & Ruf, 2005)
Movement rule	CRW: correlated random walk<br>HDM: habitat-dependent movement<br>CDM: competition-driven movement	CRW: correlated random walk<br>HDM: habitat-dependent movement	(Fletcher, 2006)
Tendency to move randomly ρ	0.3	0.3	
Mean movement distance D_{mean}	24 km (12 cells) per week	24 km (12 cells) per week	(Morelle et al., 2015)
Maximum movement distance D_{max}	84 km (42 cells) per week	84 km (42 cells) per week	(Morelle et al., 2015)
Individual weekly ranging distance $D_{m,i}$	D_i {1, D_{max}} ~ Wei(26, 1.3)	D_i {1, D_{max}} ~ Wei(26, 1.3)	
Maximal natal dispersal distance d_{disp}	3 cells (6 km)	3 cells (6 km)	(Sodeikat & Pohlmeyer, 2003)
Minimum number of dispersers N_{disp}	2 for females;<br>3 for males	2 for females;<br>3 for males	
Case fatality ratio M	{0, 0.25, 0.5, 0.75, 1}	0.5	
Mean infectious period μ	4 weeks	6 weeks	
Transmission probability β	0.022	0.022	see Appendix S2
Fertility reduction due to infection	0.625	0.625	(Kramer-Schadt et al., 2009)
Probability of prenatal infection	0.5	0.5	(Kramer-Schadt et al., 2009)
Transient period t_{trans}	1 week	1 week	(Artois et al., 2002; Moennig et al., 2003)
Period of maternal antibodies t_{anti}	12 weeks	12 weeks	(Depner, Müller, Lange, Staubach, & Teuffert, 2000)

Simulated years	12 (624 weeks)	50 (2600 weeks)	
Pathogen release	random week of the 2nd year	random week of the 2nd year	
Mean number of reproductive females	4.5	4.5	(EFSA (European Food Safety Authority), 2009; Howells & Edwards-Jones, 1997; Sodeikat & Pohlmeyer, 2003)
Habitat quality range	[0, 9]	[0, 9]	
Initial mean density	5 individuals/km^2	5 individuals/km^2	(Kramer-Schadt et al., 2009)
Initial age distribution	see Table S4	see Table S4	(Kramer-Schadt et al., 2009)
Age blur in initial individuals	± 3 weeks	± 3 weeks	
Landscape dynamic		Autocorrelated<br>Random<br>No dynamic	
Max survival time under low resources S_{max}		{5,10,15,20,25} weeks	
Level of resource mismatch		{0,25,50,75,100} %	

Table S2: Monthly reproduction probabilities

Table S2: Monthly reproduction probabilities (Boitani et al., 1995) used to stochastically determine the number of breeding females.

Month	Jan	Feb	Mar	Apr	May	Jun	Jul	Aug	Sep	Oct	Nov	Dec
Number of weeks	4	4	5	4	4	5	4	4	5	5	4	4
Reproduction probability	0.0	0.1	0.23	0.34	0.07	0.08	0.06	0.03	0.03	0.0	0.0	0.0

Table S3: Breed count distribution

Table S3: Breed count distribution (Bieber & Ruf, 2005) used to estimate litter sizes.

Litter size	0	1	2	3	4	5	6	7	8	9	10
Probability	.01306	.06915	.01629	.24994	.24994	.01629	.06915	.01910	.00343	.0004	.00002

Table S4: Initial age distribution

Table S4: Initial age distribution (Kramer-Schadt et al., 2009) used to initialize each model run.

Age (years)	1	2	3	4	5	6	7	8	9	10
Proportion	0.38	0.24	0.15	0.09	0.06	0.03	0.02	0.01	0.01	0.01

Appendix S2: Additional figures

Figure S4: Example landscape configurations

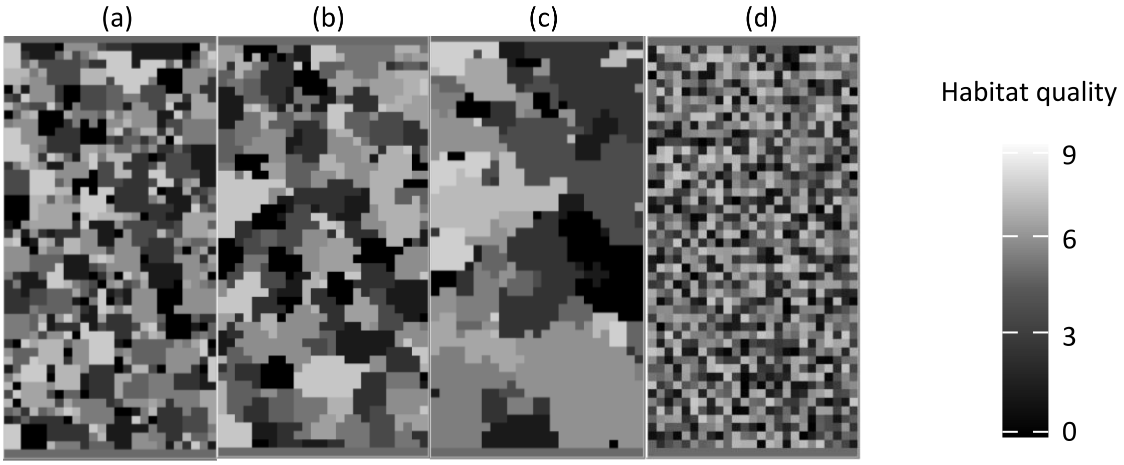

Figure S4: Example landscape configurations and habitat clustering used in the model, with (a) small clusters, (b) medium clusters, (c) large clusters of similar habitat quality and (d) randomly distributed habitat cells. The colour gradient shows the habitat quality (i.e. the maximum number of breeding females supported by the individual landscape cells).

Figure S5: Distribution of infected host individuals performing a correlated random walk (CRW) through all landscape scenarios

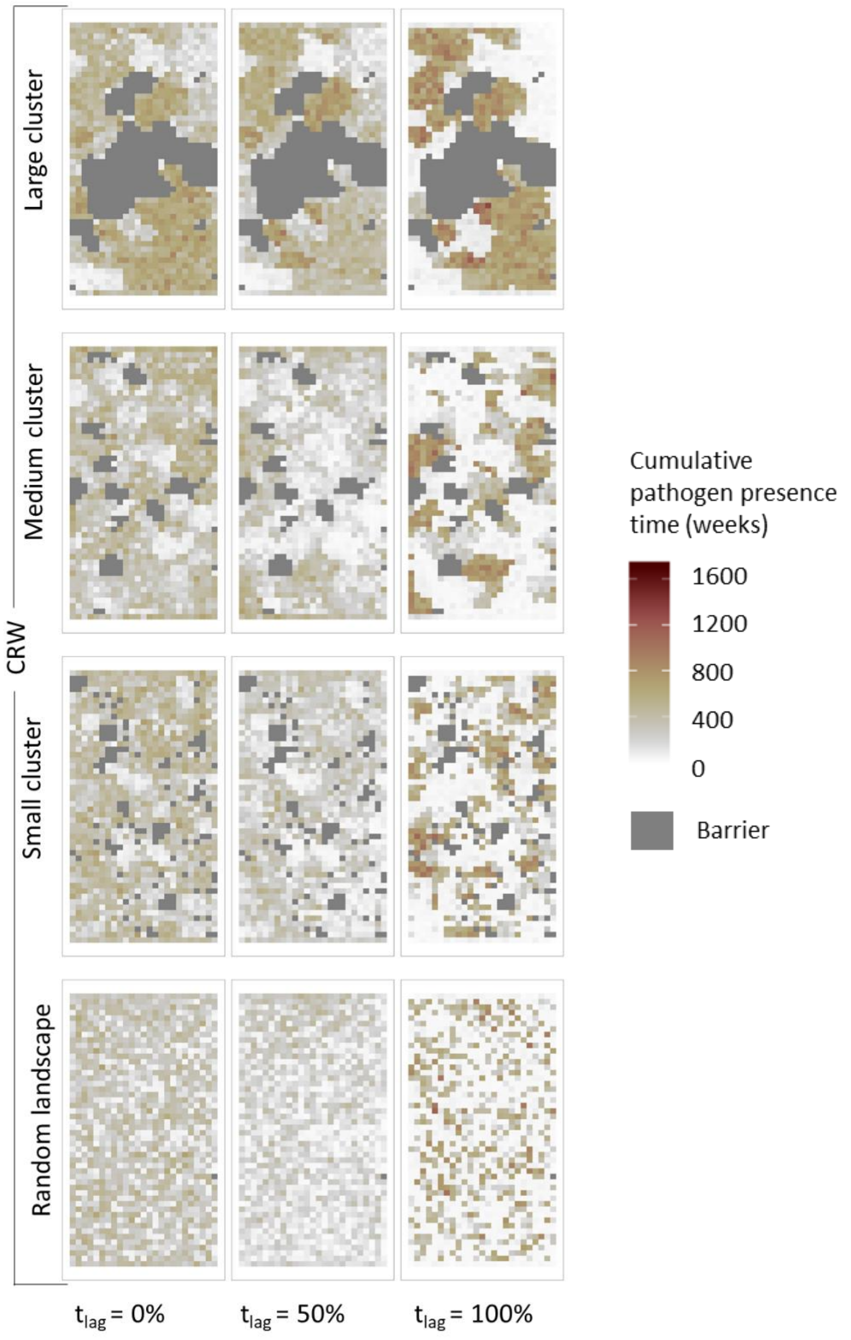

Figure S5: Spatial distribution of infected host individuals in the correlated random walk (CRW) movement scenarios throughout all landscape configurations (top to bottom: large clusters, medium clusters, small clusters, and a random configuration). The distribution is recorded as the cumulative time in weeks of infected hosts being present in the individual habitat cells at the end of a simulation run (colour gradient). Areas without any infected hosts at the end of a simulation run are further highlighted as barriers (dark grey). The distributions are further shown for three temporal shift scenarios (left to right t_{lag} 0%, t_{lag} 50% and t_{lag} 100%).

Figure S6: Distribution of infected host individuals performing habitat dependent movement (HDM) through all landscape scenarios

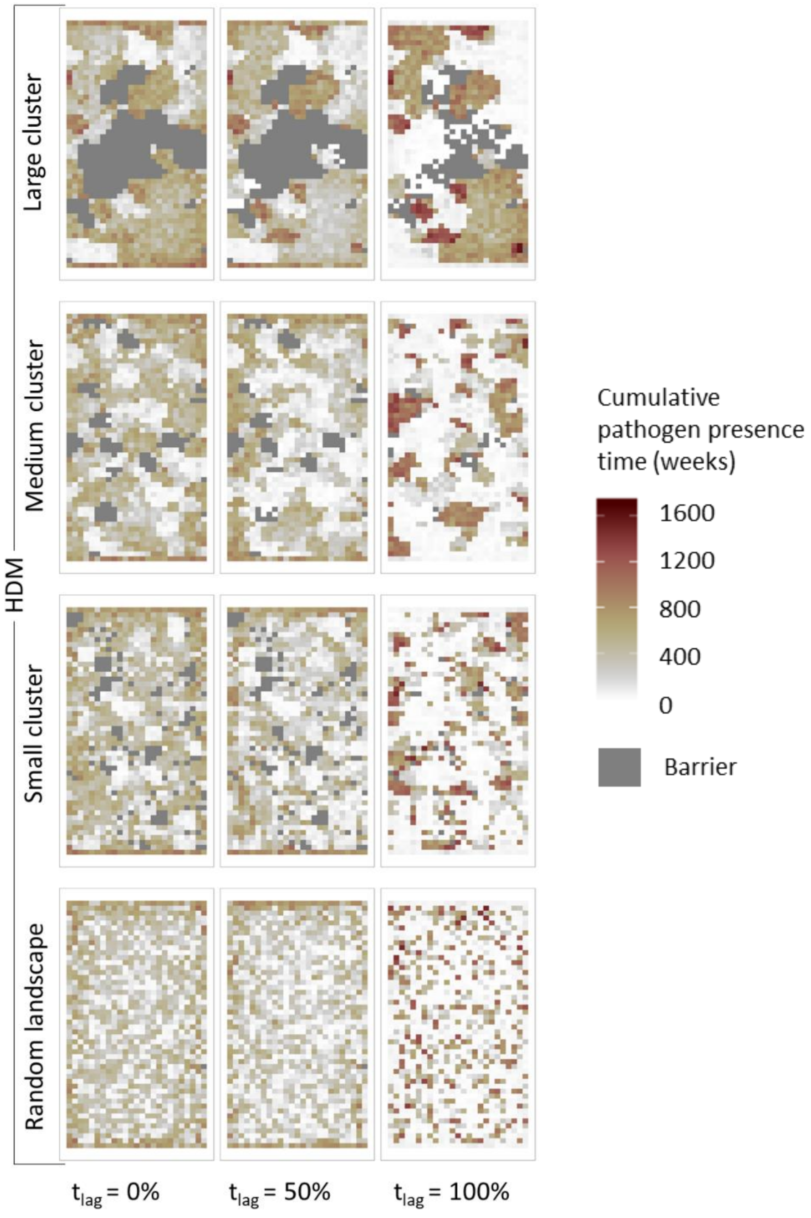

Figure S6: Spatial distribution of infected host individuals in habitat dependent movement (HDM) scenarios throughout all landscape configurations (top to bottom: large clusters, medium clusters, small clusters, and a random configuration). The distribution is recorded as the cumulative time in weeks of infected hosts being present in the individual habitat cells at the end of a simulation run (colour gradient). Areas without any infected hosts at the end of a simulation run are further highlighted as barriers (dark grey). The distributions are further shown for three temporal shift scenarios (left to right t_{lag} 0%, t_{lag} 50% and t_{lag} 100%).

Figure S7: Distribution of infected host individuals without roaming movement (neighbourhood infection) through all landscape scenarios

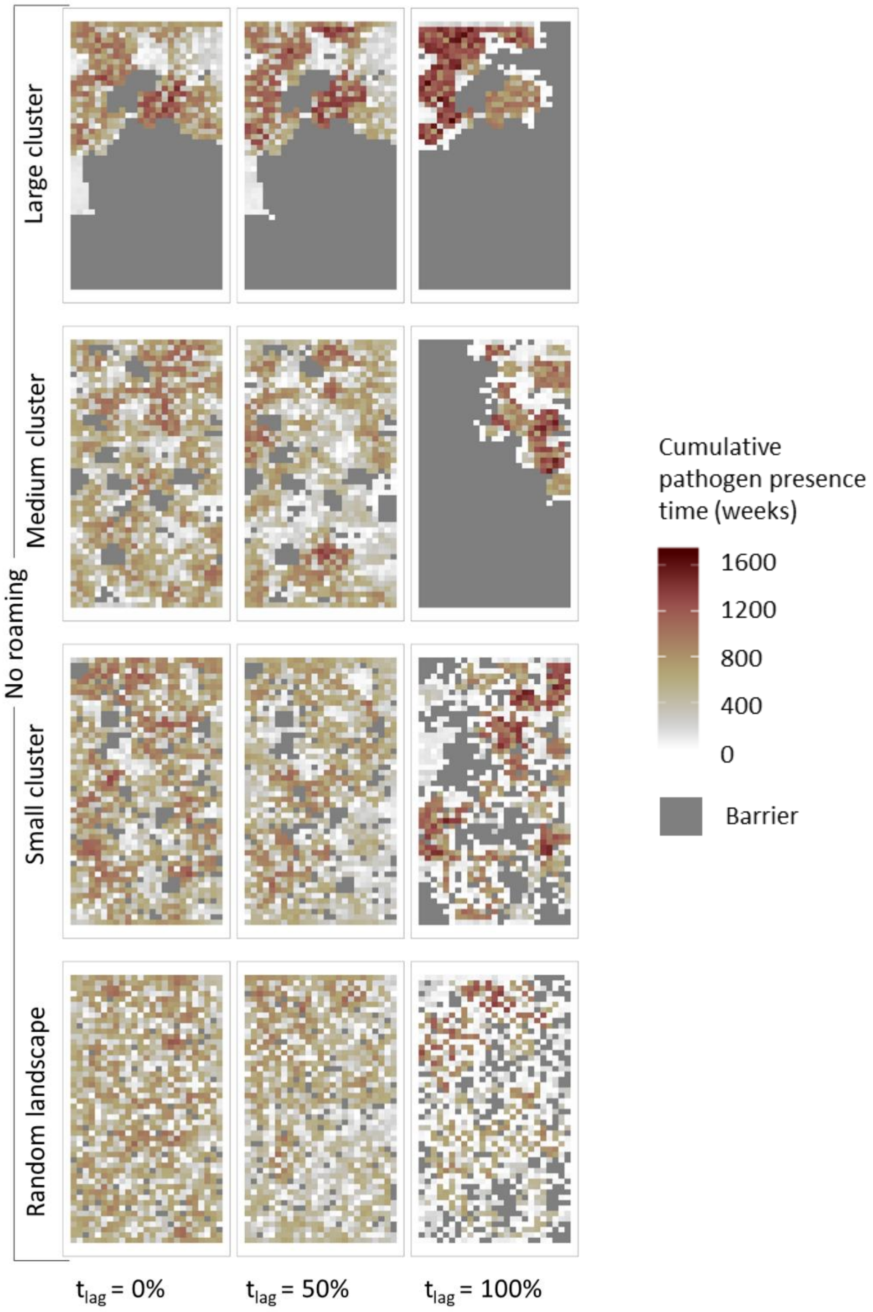

Figure S7: Spatial distribution of infected host individuals in scenarios without explicit host movement (neighbourhood infection) throughout all landscape configurations (top to bottom: large clusters, medium clusters, small clusters, and a random configuration). The distribution is recorded as the cumulative time in weeks of infected hosts being present in the individual habitat cells at the end of a simulation run (colour gradient). Areas without any infected hosts at the end of a simulation run are further highlighted as barriers (dark grey). The distributions are further shown for three temporal shift scenarios (left to right t_{lag} 0%, t_{lag} 50% and t_{lag} 100%).
